# Predicting competitive anion electrosorption on late transition metals

**DOI:** 10.1039/d5sc03757c

**Published:** 2025-08-25

**Authors:** Bolton Tran, Bryan R. Goldsmith

**Affiliations:** a University of Michigan Ann Arbor MI 48109 USA hoangtra@umich.edu

## Abstract

Investigating competitive anion electrosorption on transition metal surfaces is experimentally challenging but critical for advancing electrocatalytic and electrochemical engineering. Here, we present a rigorous computational framework for predicting anion adsorption as a function of the applied potential by combining grand canonical density functional theory (GC-DFT) with thermodynamic cycles. This approach is validated against experimental voltammograms on Pt(111) and applied to a diverse set of anions on late transition metals. Using multiple linear regression with feature importance analysis, we identify physical descriptors governing electrosorption including anion properties such as formal charge and dipole moment, and metal properties such as d-band center and atomic polarizability. We then develop a potential-dependent Langmuir adsorption model to predict competitive anion coverages under realistic electrochemical conditions. Case studies using the Langmuir model demonstrate the impact of electrolyte composition and pH on anion electrosorption trends relevant to electrocatalytic reactions such as nitrate, oxygen, and carbon dioxide reduction. Overall, this study provides a systematic and predictive framework for understanding anion electrosorption phenomena, offering insights for electrode/catalyst and electrolyte design in electrochemistry and electrocatalysis.

## Abstract 2

A theoretical framework is validated against experiments of anion electrosorption on Pt(111). Multiple linear regression model informs the physical descriptors. Potential-dependent Langmuir isotherms predict the relative anion coverage.

## Graphical abstract

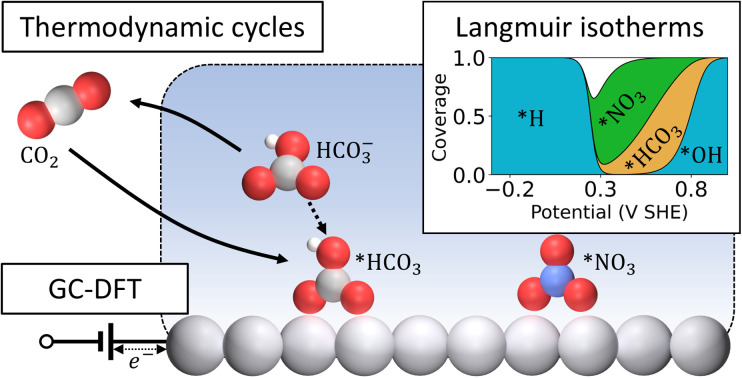

## 1 Introduction

Realistic electrochemical and electrocatalytic systems often involve multiple anions in the electrolyte. First, supporting anions are commonly used. Weakly adsorbing anions such as F^−^ and ClO_4_^−^ are used in impedance spectroscopic measurements of the electrochemical double layer.^1–4^ Buffering anions such as HCO_3_^−^, HSO_4_^−^, and H_2_PO_4_^−^ (and their acid–base conjugates) are used to mitigate pH change during reactions.^5–8^ Second, anions can be purposefully introduced to influence the activity and selectivity of electrocatalytic reactions, such as halides in CO_2_ electroreduction.^9–11^ Third, contaminating anions are often present such as in electrocatalytic treatment of nitrate-laden water, which involve NO_3_^−^ electroreduction in the presence of Cl^−^, SO_4_^2−^, HCO_3_^−^, PO_4_^3−^ as co-contaminants.^12–17^ Lastly, anion formation is ubiquitous during acid–base reactions (*e.g.*, OH^−^ is spontaneously produced from water dissociation in alkaline aqueous electrolytes).

Anions can competitively electrosorb—i.e., specifically adsorb with electron transfer—to the electrode surface, affecting surface chemistry and catalysis. Adsorbed anions can directly influence charge-transfer kinetics,^18^ modulate the co-adsorption of reaction intermediates,^9,10^ or block catalytic active sites.^13–17^ Because anions adsorb with electron transfer, the applied potentials affect their adsorption free energies. Therefore, knowledge of anion electrosorption varying with applied potential is critical for electrochemical and electrocatalytic engineering.

Detecting multiple anions electrosorbing competitively is experimentally difficult. Electroanalytical voltammetry can infer anion electrosorption from electrical currents measured at certain potential windows where the anions adsorb and transfer electrons.^19–30^ Challenges with identifying electrosorbed anions arise if the anion has multiple conjugates that co-exist (*e.g.*, H_2_PO_4_^−^ and HPO_4_^2−^),^24,26^ or if the potential windows for adsorption overlap with those of other anions or charge-transfer reactions.^27,29^ In some cases, *in situ* spectroscopic techniques can aid in species identification given discernible vibrational signals from the adsorbed anion,^5,7,31^ which is not applicable for many anions such as halides.

Density Functional Theory (DFT) calculations have been used to fill some knowledge gaps in specific anion electrosorption. DFT modeling helped identify which anion conjugates dominantly electrosorb on Pt(111) such as H_2_PO_4_^−^*versus* HPO_4_^2−^, or HSO_4_^−^*versus* SO_4_^2−^.^32–34^ DFT calculations of halides electrosorbing on Ag(111) at different coverages reproduced the broad features of experimental voltammograms.^35^ DFT also predicted the active-site blocking effects of ClO_4_^−^, NO_3_^−^, and HSO_4_^−^/SO_4_^2−^ on the activity of oxygen oxidation/reduction on Pt(111).^36^ However, past computational studies lacked the systematic and quantitative validation with experiments, as well as the identification of physical descriptors for anion electrosorption across transition metal surfaces.

In addition, many DFT studies used outdated methodologies for computing anion electrosorption free energies. First, the widely used Computational Hydrogen Electrode (CHE) method^37,38^ is an incomplete physical model of electrosorption. The model assumes that anions always fully transfer their electrons upon adsorption, that is, the electrosorption valency equals to the formal charge of the anions. For example, a CHE computation of SO_4_^2−^ adsorbing on Pt(111) automatically yields an electrosorption valency of −2.0*e*.^36^ Yet, experimental voltammetry suggests the value to be around −1.8*e*.^24^ That means SO_4_^2−^ does not fully transfer its excess electrons but retains about 0.20*e* upon adsorption to Pt(111). There are corrections to the CHE model in the form of higher-order expansions within a grand-canonical thermodynamic formalism, as well as incorporation of solvation and double-layer electrostatics.^39,40^ These corrections are important to correctly describe anion adsorption free energies as a function of the applied potential.

Second, the computation of anion adsorption free energies requires additional thermodynamic treatment to avoid systematic DFT errors for the anion aqueous-phase free energies. This treatment entails using a neutral gas-phase species as the free energy reference state by establishing a thermodynamic cycle.^14,41^ Nevertheless, many recent studies still used DFT-computed energies of aqueous-phase anions as the reference states, which greatly overestimates the adsorption free energies.^42–44^

Herein, we seek to (1) improve the rigor of DFT atomistic modeling for anion electrosorption; (2) solidify existing physical understanding of anion electrosorption on transition metals; and (3) develop a screening tool for the potential-dependent coverages of adsorbed anions on metal surfaces.

This work is organized accordingly. Section 2 outlines the computational approach using grand canonical DFT (GC-DFT) with thermodynamic cycles for predicting anion electrosorption, validated against experimental voltammograms on Pt(111). Section 3 presents a data-driven method to identify physical descriptors for anion electrosorption on transition metals. Section 4 demonstrates the prediction of anion surface coverages through a potential-dependent Langmuir model with case studies.

## 2 GC-DFT and thermodynamic cycles

The GC-DFT approach is used to compute the potential-dependent adsorption grand free energies of anions. At a target applied potential *U* (set on the SHE scale throughout this work), GC-DFT computes the grand free energy *Ω* from the Helmholtz free energy at some excess number of electrons *n*_e_ that satisfy a target Fermi energy *ε*_F_ of the electrode surface (eqn (1)).^45–47^

1 *Ω* = *E*_0_ + *E*_ZPE_ − *TS*_vib_ − *ε*_F_ × *n*_e_

The Helmholtz free energy is approximated as the ground-state energy *E*_0_ plus vibrational zero-point energy (*E*_ZPE_) and entropic corrections (*TS*_vib_). The CANDLE implicit electrolyte model was used to solvate the surface and provide counter-charges.^48^ Specific DFT details are given in Section S1.1 and Fig. S1 in the SI. To avoid erroneous DFT-computed free energy of aqueous-phase anions, thermodynamic cycles were employed.

**Fig. 1:**
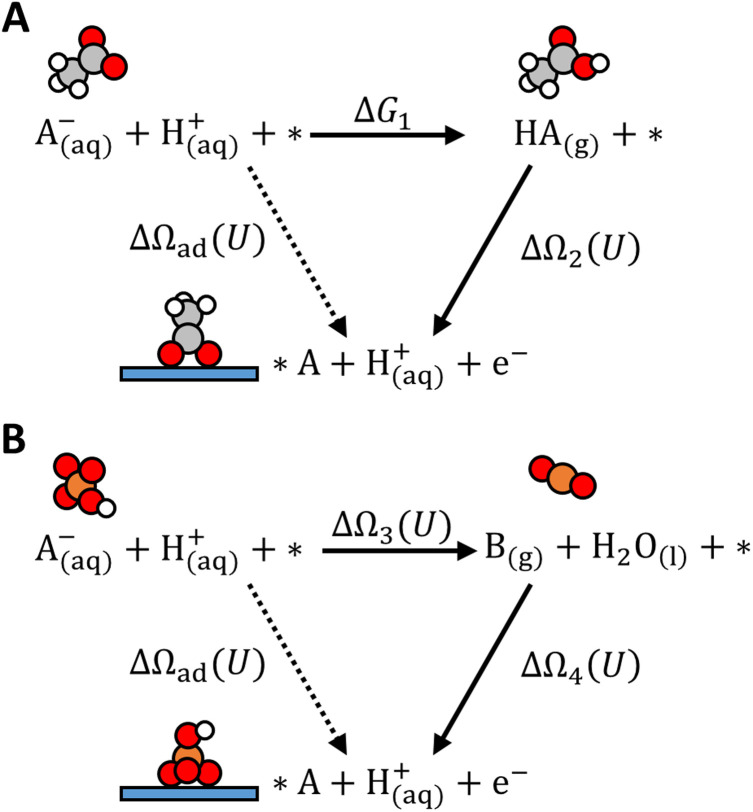
Thermodynamic cycles for computing anion adsorption free energy Δ*Ω*_ad_ (*U*). (A) Protonation path: A^−^/HA is an acid–base conjugate pair. For example, A^−^ is CH_3_COO^−^ and HA is CH_3_COOH. Δ*G*_1_ is computed from experimental p*K*_a_ and Henry's constant. (B) Redox path example: A^−^/B is a redox conjugate pair. For example, A^−^ is HSO_4_^−^ and B is SO_2_. Δ*Ω*_3_ (*U*) is computed at varying *U* from the standard redox potential. Δ*Ω*_2_ (*U*) and Δ*Ω*_4_ (*U*) are computed with GC-DFT at varying applied potential *U*. The proton–electron pair is referenced to hydrogen gas on the SHE scale.

The thermodynamic cycles establish equilibria between aqueous anions with neutral gas-phase species through a protonation path or a redox path (Fig. 1). The protonation path applies when the anions have neutral acid conjugates with known experimental values of standard Gibbs free energy of acid dissociation (*i.e.*, p*K*_a_) and Gibbs free energy of hydration (*i.e.*, Henry's constant), which add up to Δ*G*_1_ in Fig. 1A. Applicable anions for the protonation path include NO_3_^−^, RCOO^−^, HCO_3_^−^/CO_3_^2−^, HSO_3_^−^/SO_3_^2−^, and ClO_4_^−^. This thermodynamic path was previously applied for NO_3_^−^ electrosorption.^14,49^

When anions have no stable neutral acid conjugate, a redox path could be applicable if they instead have stable reduced/oxidized conjugates in the gas phase. This is the case for F^−^/Cl^−^/Br^−^ (halides) which have F_2_/Cl_2_/Br_2_ (halogens), HSO_4_^−^/SO_4_^2−^ which have SO_2_, and H_2_PO_4_^−^/HPO_4_^2−^/PO_4_^3−^ which have PH_3_. Tabulated experimental standard redox potentials for those redox pairs^50^ are used to compute Δ*Ω*_3_ in Fig. 1B at a given applied potential *U*. This path was previously applied for halides and SO_4_^2−^ electrosorption.^41^

Since aqueous electrochemical systems inevitably involve the electrosorption of protons, we also compute the potential-dependent adsorption free energy of H^+^. The thermodynamic cycle for proton electrosorption follows the classic CHE model, which involves a redox path from H^+^ to H_2_.

The thermodynamic cycles-derived formulas for potential-dependent adsorption free energies Δ*Ω*_ad_ (*U*) differ in complexity across anions. We dedicated Section S1.2 and Fig. S2–S6 in the SI detailing the thermodynamic derivation for each anion considered in this work.

**Fig. 2:**
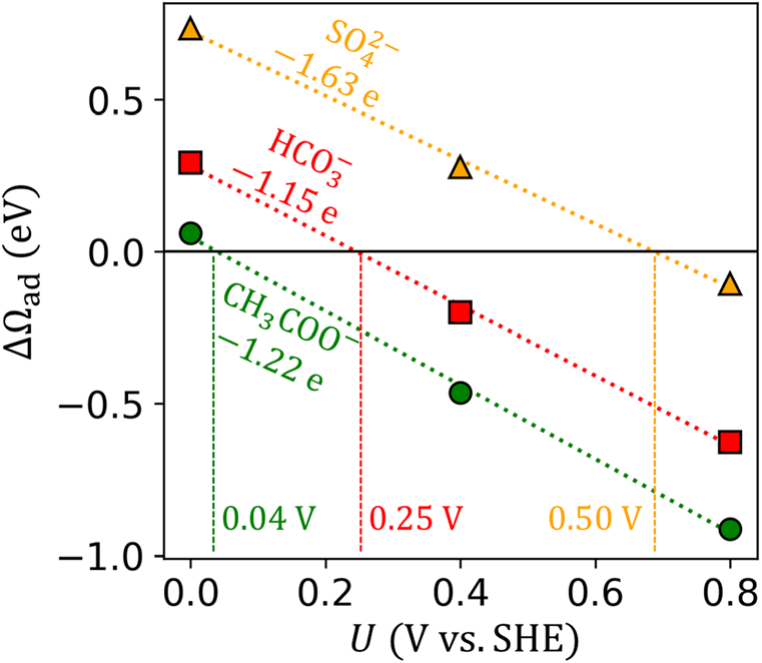
Adsorption grand free energy (Δ*Ω*_ad_) *versus* applied potential (*U* on the SHE scale) for SO_4_^2−^, HCO_3_^−^, and CH_3_COO^−^ adsorbed on Pt(111). The labeled slopes and *x*-intercept of fitted linear lines represent *γ* (unit e) and *U*^0^ (unit V), respectively.

To simplify and standardize the description of potential-dependent adsorption across many anions and metals, we extracted two key parameters for each anion-metal pair: the electrosorption valency *γ* and the standard equilibrium adsorption potential *U*^0^. The parameters were extracted from linear fitting Δ*Ω*_ad_ (*U*) at three discrete applied potentials. We picked +0.8, +0.4, and 0.0 V potentials to span the common working range of electrocatalytic experiments. We intentionally omit more negative potentials which incur weak anion binding, and more positive potentials which incur surface oxidation. The electrosorption valency—signifying the partial electron transfer as anion adsorbs—is not assumed to be an integer as in the CHE model, *i.e.*, not strictly equal to the anion formal charges. Instead, it was computed explicitly as the slope of Δ*Ω*_ad_*versus U* (Fig. 2). The standard equilibrium adsorption potential is the potential where Δ*Ω*_ad_ (*U*) is zero, *i.e.*, where equilibrium is established between adsorbed anions at some coverage with solution-phase anions at a standard concentration of 1 M. The coverage of adsorbed anions is approximated by a Langmuir model, where adsorption sites are identical and lateral interaction is omitted. The 1 M standard concentration follows the electrochemical series convention.^50^

Fig. 2 shows examples of Δ*Ω*_ad_ (*U*) computed for three anions, and the extracted *γ* and *U*^0^ values. The goodness of linear fitting for Δ*Ω*_ad_ (*U*) is presented in Table S2 in the SI.

**Fig. 3:**
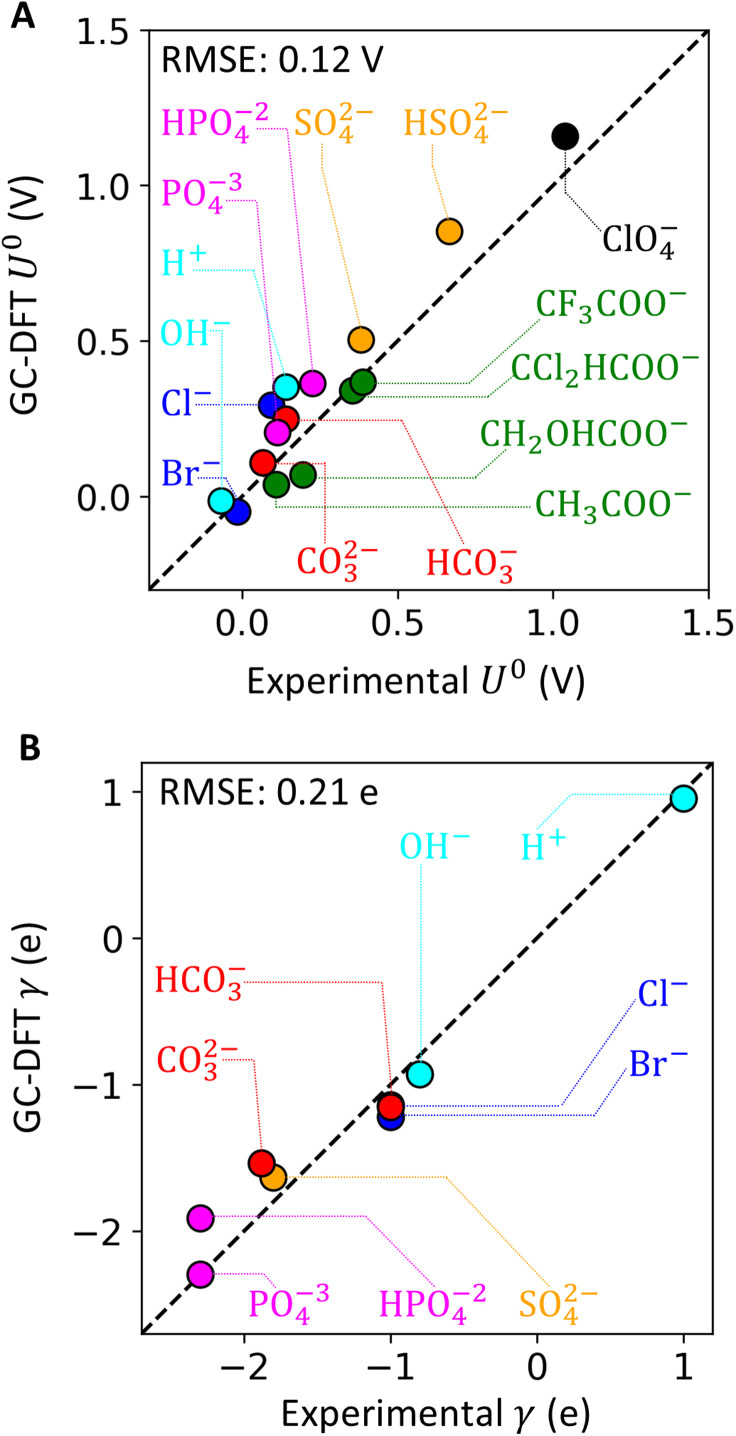
Parity plots of (A) *U*^0^ and (B) *γ* extracted from experimental voltammograms on Pt(111) surfaces *versus* obtained from GC-DFT theoretical calculations.

To validate our theoretical calculations, we compared *U*^0^ and *γ* with experimental voltammograms of different anions (and of proton) electrosorbing on Pt(111) single crystals.^20,22–25,27,28,30^ Details of experimental data extraction for each anion are presented in Section S2 and Table S3 in the SI. Fig. 3 shows good agreement between our theoretical calculations and the experimental values, with root mean squared error (RMSE) of 0.12 V for *U*^0^ and 0.21 e for *γ*.

The validation result is sensitive to the employed DFT functional, implicit electrolyte parameters, and inclusion of micro-solvation. Sensitivity analysis (Section S1.3.5 and Fig. S10 in the SI) revealed that the PBE-D3 functional^51,52^ agreed much better (lower RMSE) with experimental data compared to the RPBE functional.^53^ The computed *U*^0^ and *γ* are not as sensitive to the dielectric constant used in the implicit electrolyte. Micro-solvation^54^ may affect *U*^0^ based on the exact placement of the explicit water molecule (Fig. S11 in the SI).

This experimental validation essentially assumes a coverage-independent adsorption of anions. Specifically, we modeled anion coverages at 1/9, 2/9, or 3/9 fractional monolayer (unit monolayer corresponds to fully covered sites) for anions occupying one, two, or three Pt(111) atop sites, respectively. In voltammetry experiments, the coverages of anion on Pt(111) vary across the potential range where electrosorption occurs. Therefore, the experimental coverages at the equilibrium potential are specific to the anion, and likely at odds with the coverages used in our DFT model. This assumption is carried on to the Langmuir adsorption model used in Section 4, and partially justified at low coverages (see Section S1.3.3 and Fig. S8 in the SI).

## 3 Physical descriptors for anion electrosorption

Following the validation of our GC-DFT and thermodynamic model, we seek to identify the physical descriptors of anion electrosorption on late transition metal surfaces using a data-driven approach. Past computational work examined a limited set of anions and metal surfaces^32–36,41^ because in experiments, only a few transition metals (*e.g.*, Pt, Cu, Ag, and Au) could possibly retain metallic forms under oxidizing potentials where anions adsorb favorably. Here, we ignore electrochemical stability or direct comparison with experiments, and instead sample as many transition metals and anions as we could to search for the descriptors that encode anion electrosorption (*i.e.*, *U*^0^ and *γ*). That means expanding our dataset beyond electrochemically stable metals to include metals like Ni, Co, and Ir—known to electrochemically form oxides spontaneously.^55–57^ We model only the (111) facet, which effectively omit contributions from surface morphology.

Our dataset labels include the values of *U*^0^ and *γ*—computed using GC-DFT and thermodynamic cycles—for *n* = 107 combinations of 27 anions on 9 late transition metals. Since Pt is still the most widely used metal in controlled electrochemical experiments, the dataset is skewed toward Pt, which have data for all 27 anions. The other 8 metals (Co, Ni, Cu, Rh, Pd, Ag, Ir, and Au) only have data for 11 anions each, selected to include each of the different anion types (*i.e.*, phosphate, sulfate, carbonate, nitrate, carboxylate, and halide).

We employed a multiple linear regression (MLR) model^58^—*i.e.*, a weighted linear combination of the feature values plus a bias—trained on the physical features of anions and metals. This relatively simple model allows for easy identification and interpretation of important features. A symbolic regression model SISSO,^59^ while having greater model complexity by including non-linear operators (×÷), showed no appreciable improvement in prediction accuracy and interpretability to the MLR model (see Section S4 and Fig. S12 in the SI). Two separate MLR models were trained, one for predicting the electrosorption valency (*γ* – MLR) and one for predicting the standard equilibrium adsorption potential (*U*^0^ – MLR).

From an initial set of 11 anion features and 11 metal features (Table S4), we used feature correlation matrix (Fig. S11) to remove highly correlated features. Specifically, we removed any feature that have an *R*^2^ higher than 0.8 with another feature. We prioritized retaining features that are more physically/chemically interpretable (*e.g.*, HOMO energy of anion in radical form over that in protonated form, because anion adsorbs in radical form) and more accessible (*e.g.*, tabulated metal atomic van der Waals radius over the DFT-computed d-band width). After this step, six anion features and six metal features remained.

**Fig. 4:**
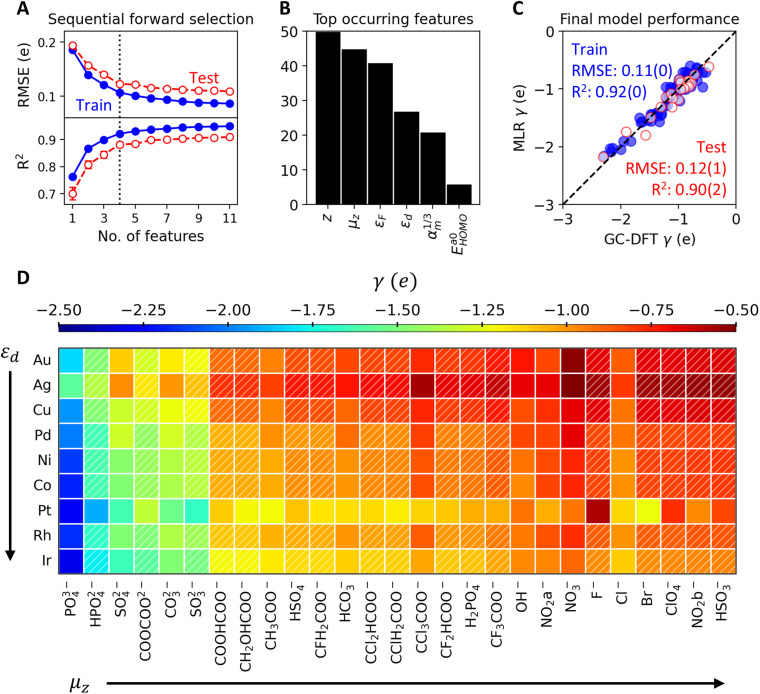
(A) Sequential forward selection applied to the *γ* – MLR model, yielding four as the optimal number of features (dotted vertical line) where RMSE and *R*^2^ no longer improves significantly with more features. (B) Histograms to count the most occurring features from 50 iterations of training the *γ* – MLR model with four features on different 80 : 20 train-test splits. (C) Parity plot for the final *γ* – MLR model trained on the four most occurring features. 5-Fold cross validation metrics (RMSE and *R*^2^) are shown as averages with standard errors in brackets. (D) Dataset matrix with electrosorption valency (*γ*) values computed from GC-DFT (solid fill) or from the optimized *γ* – MLR model (diagonal hatch). The matrix is ordered by increasing *μ*_*z*_ for the anions and *ε*_d_ for the metals, following the arrow directions.

Next, we performed sequential forward selection (SFS) to further optimize the number of features while avoiding overfitting. The SFS algorithm starts by looping through the features to find one that yields the lowest RMSE when evaluated on the test set of an 80 : 20 train-test split. The algorithm then adds to the model a second feature that yields the lowest RMSE, and keeps sequentially adding more features using similar criteria afterwards. Fig. 4A shows the RMSE going down and the *R*^2^ for label-prediction parity going up as the number of features increases for the *γ* – MLR model. The dataset was bootstrapped over 50 iterations of different 80 : 20 train-test splits to obtain small statistical error bars in Fig. 4A. While no overfitting is apparent (when training RMSE goes down while test RMSE goes up with the number of features), we selected four as the optimal number of features for the *γ* – MLR model, since the RMSE and *R*^2^ no longer improve significantly with more features.

Bootstrapping data in the SFS algorithm furthermore allows for counting the top occurring features for the *γ* – MLR model (Fig. 4B). We picked the four most occurring features to be the descriptors that encode the electrosorption valency *γ*. A final 5-fold cross validation was performed on the *γ* – MLR model with these four top-occurring features (Fig. 4C), yielding test-set RMSE of 0.12 ± 0.01*e* and *R*^2^ of 0.90 ± 0.02.

The same SFS workflow was applied to a separate *U*^0^ – MLR model for predicting the standard equilibrium adsorption potential (Fig. S13 in the SI). The optimal number of features for predicting *U*^0^ was six. A 5-fold cross validation performed on the final *U*^0^ – MLR model yielded test-set RMSE of 0.21 ± 0.02 V and *R*^2^ of 0.75 ± 0.05.

Table 1 layouts the identified descriptors for *γ* and *U*^0^. Coincidentally, the descriptors for *γ* and *U*^0^ do not overlap, and there are equal numbers of anion descriptors and metal descriptors for each quantity. The coefficient signs +/− indicate correlation/anticorrelation between the features and the labels. The final MLR equations for *γ* and *U*^0^ are shown in Section S5.3 in the SI. Next, we briefly discuss how the identified descriptors physically associate with *γ* and *U*^0^.

**Table 1:** Symbols, coefficient signs (+/−), and description of the identified descriptors for predicting *γ* and *U*^0^ with MLR models

Target	Anion/Metal	Symbol (+/−)	Feature description
*γ*	Anion	*z* (+)	Anion formal charge
		*μ*_*z*_ (+)	Surface-normal dipole moment in adsorbed state
	Metal	*ε*_F_ (+)	Fermi energy of (111) slab in implicit electrolyte
		*ε*_d_ (−)	d-Band center
*U* ^0^	Anion	*E* ^a0^_HOMO_ (−)	HOMO energy of anion in radical form
		*E* ^a1^_EA_ (+)	Electron affinity to create −1 charge
		Δ*E*^dis^_H_ (+)	Energy of homolytic proton dissociation
	Metal	*E* ^m^_EA_ (+)	Atomic electron affinity
		*α*_m_^1/3^ (−)	Cubic root of atomic polarizability
		*r*_vdw_ (+)	Atomic VdW radius

The descriptors of electrosorption valency *γ* are related to the electron transfer when the anion adsorbs. First, *γ* positively correlates with anions' formal charges *z* and molecular dipole in adsorbed state *μ*_*z*_. This indicates that more electron transfer (more negative *γ*) corresponds with anions having more negative formal charges (*e.g.*, SO_4_^2−^ over HSO_4_^−^) and smaller molecular dipoles in the adsorbed state (Fig. 4D). The relationship between adsorbate dipole moment and charge-transfer has been established in past work.^60–64^ Second, *γ* positively correlates with the Fermi energy *ε*_F_ of the metal, indicating that metals with more stable electrons (more negative *ε*_F_) allow for more electrons transferring from the adsorbed anions (more negative *γ*). Third, *γ* negatively correlates with the d-band center *ε*_d_ of the metal, suggesting less filled d-bands (more positive *ε*_d_) accept more electrons from the adsorbed anions (more negative *γ*).^64^

By partitioning *γ* with respect to the anion formal charges *z* (Fig. S14), we also observed that anions with more negative *z* retain more negative partial charges when they adsorb. Specifically, mono-, di-, and tri-valent anions retain on average 0*e*, −0.5*e*, and −1*e* partial charges, respectively. Therefore, the CHE model assumption that anions transfer all of its excess electrons upon adsorption would be especially wrong for divalent/trivalent anions.

The descriptors for standard equilibrium adsorption potential *U*^0^ encode the binding strength of anions to the metal surfaces. The most intuitive anion descriptor is the energy of homolytic proton dissociation, where a more negative Δ*E*^dis^_H_—harder to break the anion-proton bond—correlates with a more negative *U*^0^—more negative bias needed to desorb the anion from the surface, both suggesting anions' strong tendency to form covalent bonds.^30,34^ For metals, the atomic VdW radii and atomic polarizability may encode the short-range repulsion and dispersion.^65,66^

## 4 Potential-dependent Langmuir isotherms

To quickly inform the electrode/electrolyte/pH design in electrocatalytic experiments, we constructed a potential-dependent Langmuir model to predict the competitive anion adsorption on metal surfaces at different applied potentials. The idea is that for any given set of anions with defined bulk concentrations, solution pH, and transition metal electrode, the model could predict the relative coverages of electrosorbed anions (and the protons) at varying applied potentials.

The model establishes adsorption equilibria at each applied potential between solution-phase anions and adsorbed anions, and acid–base equilibria between solution-phase anions and their conjugates, but not redox equilibria between redox pairs. That is, this Langmuir model predicts the surface coverages of anions at a timescale where redox kinetics (*i.e.*, charge-transfer reaction) are much slower than adsorption kinetics and acid–base kinetics.^67,68^

In brief, the Langmuir isotherms are computed by solving two systems of equations. The first linear equations system solves the acid–base equilibria for the solution-phase concentration of each conjugate, which require pH and total acid concentration as input constants. The second non-linear equation system solves the potential-dependent Langmuir adsorption equilibria for the surface coverage of each anion, which require the *γ* and *U*^0^ values for each anion-metal pair as computed using the GC-DFT and thermodynamic cycle method. A full derivation for this potential-dependent Langmuir model is outlined in Section S6 of the SI.

A Langmuir adsorption model makes inherent assumptions. First, it assumes no adsorbate–adsorbate interactions. Second, it uses a mean-field approximation of adsorption sites, which means the absolute coverages can be up to 1.0, *i.e.*, all the sites are fully covered. A molecular picture of anion adsorption on (111) facets does not quite allow for a fully covered surface for many anions. Therefore, the coverages should be interpreted in relative terms between the adsorbed species.

**Fig. 5:**
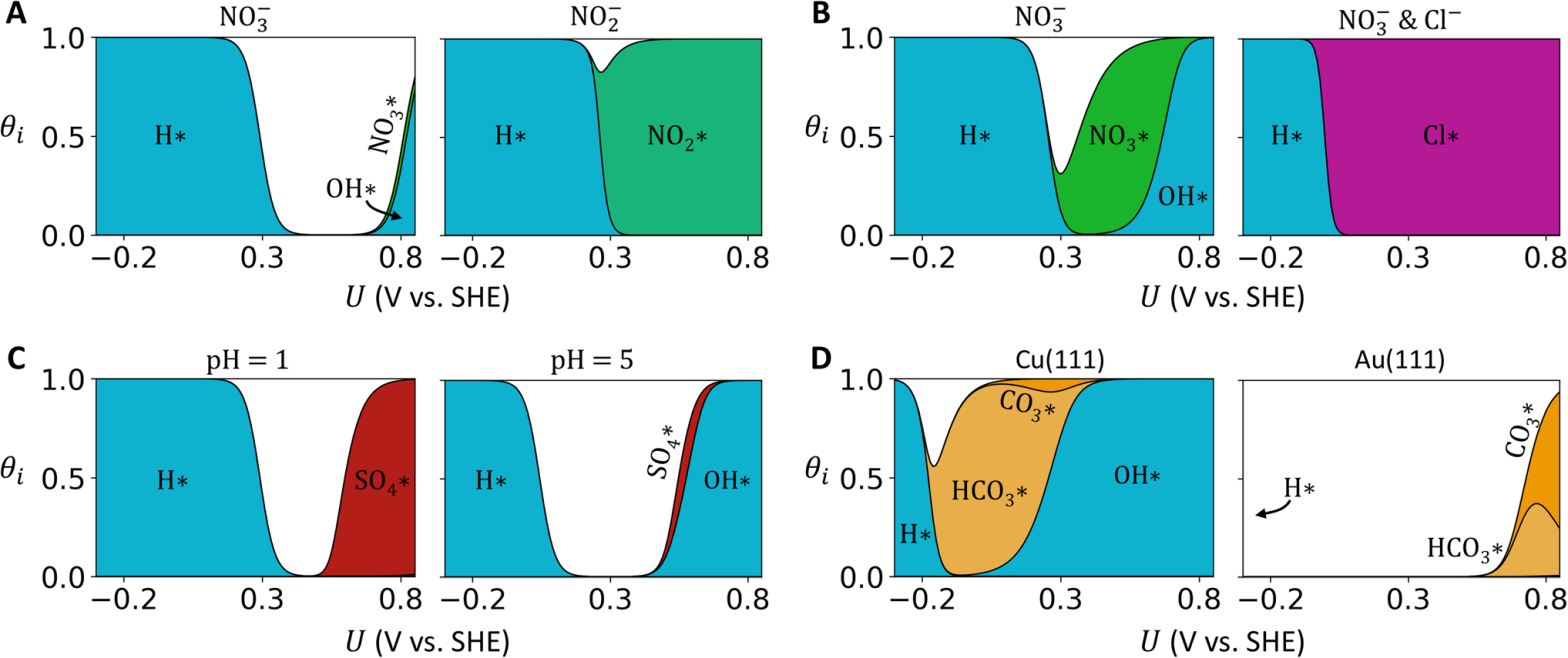
Potential-dependent Langmuir isotherms for four case studies: (A) Pt(111), pH 1, 100 mM ClO_4_^−^, 10 mM NO_3_^−^ (left) or 10 mM NO_2_^−^ (right). (B) Cu(111), pH 1, 100 mM ClO_4_^−^, 50 mM NO_3_^−^ (left) or 50 mM NO_3_^−^ & 10 mM Cl^−^ (right). (C) Pt(111), 100 mM SO_4_^2−^/HSO_4_^−^, pH 1 (left) or pH 5 (right). (D) pH 8.3, 100 mM CO_3_^2−^/HCO_3_^−^ on Cu(111) (left) or 500 mM CO_3_^2−^/HCO_3_^−^ on Au(111) (right).

Fig. 5 presents the potential-dependent Langmuir isotherms (*i.e.*, surface coverage *θ*_*i*_ of anion *i vs.* applied potential *U* in SHE scales) for four case studies. While containing no information about reaction mechanisms and kinetics, these isotherms can indirectly inform about the feasibility of surface reactions through observing the adsorption of poisoning anions, or the co-adsorption of reactants at certain potential windows.

In the first case study (Fig. 5A), we corroborate experimental results for the electrocatalytic reduction of NO_3_^−^ and NO_2_^−^ on Pt. At an onset potential of 0.2 V on a Pt(111) catalyst, da Silva and co-workers observed the reductive current of NO_2_^−^ to be almost an order of magnitude larger than that of NO_3_^−^.^29^ Our Langmuir isotherms at the same experimental conditions (pH and anion concentrations) predict the coverage of NO_3_^−^ (Fig. 5A left) to drop at a more positive potential than that of NO_2_^−^ (Fig. 5A right). Furthermore, only the adsorption of NO_2_^−^ greatly overlaps with the adsorption of protons (H^+^) near 0.2 V, where reductive current onsets. The co-adsorption of H^+^ with NO_2_^−^ (which is absent for NO_3_^−^) potentially facilitates hydrogenation elementary steps involved in the catalytic reduction mechanism.^14,29^

The poor catalytic performance of Pt for NO_3_^−^ reduction (NRR) relative to other metal catalysts (*e.g.*, Cu or Rh) is generally known and attributed to the weaker binding of NO_3_^−^ on Pt.^14,69^ From the identified descriptors (Table 1), one may partially explain this adsorption and catalysis trend by seeing that Pt has a higher atomic VdW radius (2.14 Å) than Cu (1.96 Å) and Rh (2.10 Å), leading to stronger repulsion against adsorbed NO_3_^−^.

In the second case study (Fig. 5B), we examine the poisoning effect of Cl^−^ on Cu-catalyzed NRR. Butcher and Gewirth observed an onset potential for NRR on Cu(111) at about 0.2 V, which then shifted to −0.2 V in the presence of Cl^−^ anion.^70^ They attributed this to Cl^−^ competitively adsorbing and poisoning adsorption sites for NO_3_^−^. The Langmuir isotherms at the same experimental conditions show co-adsorption of NO_3_^−^ and H^+^ near the NRR onset potential of 0.2 V (Fig. 5B left), at which adsorbed Cl^−^ would then effectively poison the active sites (Fig. 5B right). However, the model fails to explain how NRR still takes place at −0.2 V, where it predicts that proton H^+^ completely displaces adsorbed Cl^−^ and no NO_3_^−^ adsorbs. One possible explanation is that some form of non-Langmuir adsorption might have taken place (*e.g.*, co-adsorbates interact and form clusters/islands), allowing for NO_3_^−^ to still adsorb and reduce at −0.2 V where Cl^−^ poison has been alleviated.

In the third case study (Fig. 5C), we demonstrate how increases in pH can potentially alleviate SO_4_^2−^ poisoning for Pt-catalyzed oxygen reduction reaction (ORR). Experiments by Kamat and co-workers found that SO_4_^2−^ decreases the ORR current density on a Pt disk at a pH of 1 below the onset potential at about 0.8–0.9 V.^36^ They attributed the lowered activity to SO_4_^2−^ adsorbing competitively with OH^−^, which is a reaction intermediate for ORR. At the same pH of 1, the model consistently predicts SO_4_^2−^ to dominate the adsorption sites below 0.8 V (Fig. 5C left). A higher solution pH of 5 increases the abundance of solution-phase OH^−^ anion, at which the model predicts OH^−^ to out-competes and displaces SO_4_^2−^ on the surface (Fig. 5C right).

In the fourth and final case study (Fig. 5D), we examine the adsorption of HCO_3_^−^ and CO_3_^2−^ during CO_2_ reduction reaction (CO_2_RR) on Cu and Au catalysts. On Cu, Zhu and co-workers found vibrational signals of adsorbed HCO_3_^−^/CO_3_^2−^ (1544–1517 cm^−1^) disappearing while scanning applied potentials from 0.6 V to 0.1 V.^7^ On Au, Dunwell and co-workers found the same vibrational modes (∼1460 cm^−1^) disappearing while scanning potentials from 1.5 V to 0.8 V.^5^ They respectively observed slight blue-shifts in frequency at 0.3 V on Cu and 1.0 V on Au, which earlier work attributed to adsorbed HCO_3_^−^ displacing adsorbed CO_3_^2−^.^28^ Consistent with above spectroscopic evidences, our isotherms predict the coverage of HCO_3_^−^ increases as coverage of CO_3_^2−^ decreases at about 0.3 V on Cu(111) (Fig. 5D left) and 0.9 V on Au(111) (Fig. 5D right).

In the catalytic context of CO_2_RR, the isotherms are also consistent with the different product selectivity on Cu and Au. It is well known that Cu reduces CO_2_ to hydrogenated products (*e.g.*, CH_4_, CH_3_OH) while Au reduces CO_2_ to CO.^71,72^ Furthermore, Zhu and Dunwell found vibrational signals of CO intermediate appearing at potentials coinciding with when HCO_3_^−^ desorbs, *i.e.*, below 0.0 V on Cu, and below 0.8 V on Au.^5,7^ On Cu, the isotherms predict protons to adsorb at about 0.0 V (Fig. 5D left). Under the assumption that CO indeed adsorbs on Cu also at 0.0 V, the overlap in coverages of protons and CO is conducive to the hydrogenation of CO. Conversely on Au, the isotherms predict protons to only adsorb at potentials well below where CO adsorbs supposedly at 0.8 V (Fig. 5D left), which is consistent with the lack of hydrogenated products.

## 5 Conclusion

While inferring competitive anion electrosorption is challenging in experiments, DFT can provide helpful predictions given rigorous model construction and validation. We presented here a grand-canonical DFT approach used in conjunction with thermodynamic cycles to account for the applied potentials and the free energy of solution-phase anions. We validated the model against experimental voltammograms of Pt(111) with different electrolytic anions. This computational approach can be transferrable to study anion electrosorption on more complex electrodes/catalysts such as oxides, alloys, and single-atom catalysts.

We applied the multiple linear regression (MLR) models on a diverse dataset of anions electrosorbing on transition metal (111) surfaces to identify physical descriptors encoding the electrosorption valency and the standard equilibrium adsorption potential. We recovered the association of adsorbate dipole moment and metal d-band with the electrosorption valency, as well as adsorbate-metal covalent binding strength with the standard equilibrium adsorption potential. Furthermore, our dataset of (111) facets of late transition metals and small anions provides a controlled benchmark to explore for similar/deviating physical descriptors for electrosorption on more complex catalyst materials, surface facets, or with bulky organic anions and cations electrosorbing.

We constructed a potential-dependent Langmuir model and showcased how information about the (in)feasibility of electrocatalytic reactions could be derived through case studies. This adsorption model can be further expanded to electrocatalytic materials more complex than single-crystal transition metals by accounting for multiple sites (facets, reconstructed/oxidized surfaces) and coverage-dependent adsorption.

## Author contributions

B. Tran: conceptualization, data curation, formal analysis, methodology, visualization, writing-original draft; B. R. Goldsmith: conceptualization, validation, funding acquisition, project administration, supervision, writing-review & editing.

## Conflicts of interest

There are no conflicts to declare.

## Supplementary Material

SC-OLF-D5SC03757C-s001

## Data Availability

Optimized structures, DFT files, data tables, and Python codes for analysis are available in an open-access repository at https://doi.org/10.5281/zenodo.16619520. Supplementary information: GC-DFT calculation details; model sensitivity tests; derivation of thermodynamic cycles; extraction of experimental data; dataset and feature details; symbolic regression model; additional MLR model results; and Langmuir model derivation. See DOI: https://doi.org/10.1039/d5sc03757c.

## References

[cit1] Grahame D. C. (1954). J. Am. Chem. Soc..

[cit2] Valette G. (1982). J. Electroanal. Chem..

[cit3] Pajkossy T., Wandlowski T., Kolb D. M. (1996). J. Electroanal. Chem..

[cit4] Pajkossy T., Kolb D. (2001). Electrochim. Acta.

[cit5] Dunwell M., Lu Q., Heyes J. M., Rosen J., Chen J. G., Yan Y., Jiao F., Xu B. (2017). J. Am. Chem. Soc..

[cit6] Wuttig A., Yoon Y., Ryu J., Surendranath Y. (2017). J. Am. Chem. Soc..

[cit7] Zhu S., Jiang B., Cai W. B., Shao M. (2017). J. Am. Chem. Soc..

[cit8] Resasco J., Lum Y., Clark E., Zeledon J. Z., Bell A. T. (2018). Chemelectrochem.

[cit9] Cho M., Song J. T., Back S., Jung Y., Oh J. (2018). ACS Catal..

[cit10] Huang Y., Ong C. W., Yeo B. S. (2018). ChemSusChem.

[cit11] Chen W., Zhang L. L., Wei Z., Zhang M. K., Cai J., Chen Y. X. (2023). Phys. Chem. Chem. Phys..

[cit12] Su C., Puls R. W. (2004). Environ. Sci. Technol..

[cit13] Wang Y., Qu J., Liu H. (2007). J. Mol. Catal. A: Chem..

[cit14] Richards D., Young S. D., Goldsmith B. R., Singh N. (2021). Catal. Sci. Technol..

[cit15] González D., Baeza J., Calvo L., Gilarranz M. (2023). J. CO_2_ Util..

[cit16] Sacco N. A., Zoppas F. M., Beltrame T. F., Miró E. E., Marchesini F. A. (2023). Environ. Sci. Pollut. Res..

[cit17] Fan J., Arrazolo L. K., Du J., Xu H., Fang S., Liu Y., Wu Z., Kim J. H., Wu X. (2024). Environ. Sci. Technol..

[cit18] Agarwal H., Florian J., Goldsmith B. R., Singh N. (2021). Cell Rep. Phys. Sci..

[cit19] Herrero E., Mostany J., Feliu J. M., Lipkowski J. (2002). J. Electroanal. Chem..

[cit20] Mostany J., Herrero E., Feliu J. M., Lipkowski J. (2003). J. Electroanal. Chem..

[cit21] Berna A., Rodes A., Feliu J. M., Illas F., Gil A., Clotet A., Ricart J. M. (2004). J. Phys. Chem. B.

[cit22] Garcia-Araez N., Climent V., Herrero E., Feliu J., Lipkowski J. (2005). J. Electroanal. Chem..

[cit23] Garcia-Araez N., Climent V., Herrero E., Feliu J., Lipkowski J. (2006). J. Electroanal. Chem..

[cit24] Garcia-Araez N., Climent V., Rodriguez P., Feliu J. M. (2008). Electrochim. Acta.

[cit25] Mostany J., Martínez P., Climent V., Herrero E., Feliu J. M. (2009). Electrochim. Acta.

[cit26] Gisbert R., García G., Koper M. T. (2010). Electrochim. Acta.

[cit27] Attard G. A., Brew A., Hunter K., Sharman J., Wright E. (2014). Phys. Chem. Chem. Phys..

[cit28] Martínez-Hincapié R., Berná A., Rodes A., Climent V., Feliu J. M. (2016). J. Phys. Chem. C.

[cit29] da Silva K. N., Soffiati G., da Silva E. Z., San-Miguel M. A., Sitta E. (2022). New J. Chem..

[cit30] Hasan M. H., McCrum I. T. (2024). Angew. Chem., Int. Ed..

[cit31] Su Z., Climent V., Leitch J., Zamlynny V., Feliu J. M., Lipkowski J. (2010). Phys. Chem. Chem. Phys..

[cit32] Savizi I. S. P., Janik M. J. (2011). Electrochim. Acta.

[cit33] Jinnouchi R., Hatanaka T., Morimoto Y., Osawa M. (2012). Phys. Chem. Chem. Phys..

[cit34] Yeh K. Y., Restaino N. A., Esopi M. R., Maranas J. K., Janik M. J. (2013). Catal. Today.

[cit35] Hörmann N. G., Reuter K. (2021). J. Chem. Theory Comput..

[cit36] Kamat G. A., Zamora Zeledón J. A., Gunasooriya G. T. K., Dull S. M., Perryman J. T., Nørskov J. K., Stevens M. B., Jaramillo T. F. (2022). Commun. Chem..

[cit37] Nørskov J. K., Rossmeisl J., Logadottir A., Lindqvist L., Kitchin J. R., Bligaard T., Jónsson H. (2004). J. Phys. Chem. B.

[cit38] Chan K., Nørskov J. K. (2015). J. Phys. Chem. Lett..

[cit39] Hörmann N. G., Marzari N., Reuter K. (2020). npj Comput. Mater..

[cit40] Agrawal N., Wong A. J. W., Maheshwari S., Janik M. J. (2024). J. Catal..

[cit41] Granda-Marulanda L. P., McCrum I. T., Koper M. T. M. (2021). J. Phys.: Condens. Matter.

[cit42] Jing H., Long J., Li H., Fu X., Xiao J. (2023). Chin. J. Catal..

[cit43] Wang F., Zhao H., Zhang G., Zhang H., Han X., Chu K. (2024). Adv. Funct. Mater..

[cit44] Hong Q. L., Zhong W., He K. Y., Sun B., Ai X., Xiao X., Chen Y., Yu Xia B. (2024). Adv. Sustainable Syst..

[cit45] Sundararaman R., Goddard W. A., Arias T. A. (2017). J. Chem. Phys..

[cit46] Hörmann N. G., Andreussi O., Marzari N. (2019). J. Chem. Phys..

[cit47] Goddard W. A., Song J. (2023). Top. Catal..

[cit48] Sundararaman R., Goddard W. A. (2015). J. Chem. Phys..

[cit49] Jing H., Long J., Li H., Fu X., Xiao J. (2023). ACS Catal..

[cit50] Bratsch S. G. (1989). J. Phys. Chem. Ref. Data.

[cit51] Perdew J. P., Chevary J. A., Vosko S. H., Jackson K. A., Pederson M. R., Singh D. J., Fiolhais C. (1992). Phys. Rev. B: Condens. Matter Mater. Phys..

[cit52] Grimme S., Antony J., Ehrlich S., Krieg H. (2010). J. Chem. Phys..

[cit53] Hammer B., Hansen L. B., Nørskov J. K. (1999). Phys. Rev. B: Condens. Matter Mater. Phys..

[cit54] Clabaut P., Schweitzer B., Götz A. W., Michel C., Steinmann S. N. (2020). J. Chem. Theory Comput..

[cit55] Badawy W. A., Al-Kharafi F. M., Al-Ajmi J. R. (2000). J. Appl. Electrochem..

[cit56] Hall D. S., Bock C., MacDougall B. R. (2013). J. Electrochem. Soc..

[cit57] Cherevko S., Geiger S., Kasian O., Mingers A., Mayrhofer K. J. (2016). J. Electroanal. Chem..

[cit58] Jobson J. D. , Applied Multivariate Data Analysis, Springer, New York, 1991, ch. 4, pp. 219–398

[cit59] Ouyang R., Curtarolo S., Ahmetcik E., Scheffler M., Ghiringhelli L. M. (2018). Phys. Rev. Mater..

[cit60] Leung C., Kao L., Su S., Feng J., Chan T. (2003). Phys. Rev. B: Condens. Matter Mater. Phys..

[cit61] Rusu P. C., Brocks G. (2006). J. Phys. Chem. B.

[cit62] Otero R., Vázquez de Parga A. L., Gallego J. M. (2017). Surf. Sci. Rep..

[cit63] Wang X., Ye S., Hu W., Sharman E., Liu R., Liu Y., Luo Y., Jiang J. (2020). J. Am. Chem. Soc..

[cit64] Sweeney D. M., Tran B., Goldsmith B. R. (2025). Commun. Chem..

[cit65] Adamovic I., Gordon M. S. (2005). Mol. Phys..

[cit66] Olasz A., Vanommeslaeghe K., Krishtal A., Veszpŕmi T., Van Alsenoy C., Geerlings P. (2007). J. Chem. Phys..

[cit67] Fogler H. S. , Goldsmith B. R., Nikolla E. and Singh N., Elements of Chemical Reaction Engineering, Pearson Education, 7th edn, 2025, ch. 10, p. 447

[cit68] Motagamwala A. H., Dumesic J. A. (2021). Chem. Rev..

[cit69] Shibata M., Yoshida K., Furuya N. (1998). J. Electrochem. Soc..

[cit70] Butcher D. P., Gewirth A. A. (2016). Nano Energy.

[cit71] Hori Y., Wakebe H., Tsukamoto T., Koga O. (1994). Electrochim. Acta.

[cit72] Peterson A. A., Nørskov J. K. (2012). J. Phys. Chem. Lett..

