# Supplementary material for: Predicting competitive anion electrosorption on late transition metals
Source: Chem Sci. 2025 Aug 25;16(37):17325–33. doi: 10.1039/d5sc03757c (PMC12396136; doi:10.1039/d5sc03757c)
Supplement: SC-016-D5SC03757C-s001 [file SC-016-D5SC03757C-s001.pdf]

Supplementary Information (SI) for Chemical Science.
This journal is © The Royal Society of Chemistry 2025

Predicting Competitive Anion Electrosorption on Late Transition Metals

Supplementary Information

Bolton Tran* and Bryan R. Goldsmith

Department of Chemical Engineering, University of Michigan, Ann Arbor, MI 48109, USA

E-mail: hoangtra@umich.edu

Contents

S1 Computational methods **2**

S1.1 GC-DFT calculation details 2

S1.2 Derivation of thermodynamic cycles 4

S1.3 Sensitivity analysis of parameters in GC-DFT model 10

S1.3.1 Goodness of fit of $\Delta\Omega_{\text{ad}}$ vs. U 10

S1.3.2 Slab construction 11

S1.3.3 Coverage 12

S1.3.4 Vibrational correction vs. potential 13

S1.3.5 DFT functionals and implicit solvent dielectric constants 14

S1.3.6 Micro-solvation with explicit water 15

S2 Extracting results from experimental voltammograms **16**

S3 Anion-metal dataset **18**

S3.1 Description and sources of features 18

S3.2 Feature correlation matrix 19

S4 Symbolic regression model **20**

S5 Multiple linear regression (MLR) model **22**

S5.1 MLR results for U^0 22

S5.2 Retained charge analysis 23

S5.3 Anion MLR equations 23

S6 Potential-dependent Langmuir model **24**

References **26**

S1 Computational methods

S1.1 GC-DFT calculation details

DFT calculations were performed using the JDFTx software package.[1] The Perdew-Burke-Ernzerhof functional (PBE)[2] or its revised version (RPBE)[3] were used with the GBRV pseudopotentials.[4] The D3 dispersion correction (non-damping) was used with the PBE functional.[5] The kinetic energy cutoffs for the wavefunction and charge density were 20 and 100 Ha, respectively. A Monkhorst-Pack k-point grid of 4×4×1 was used to sample the Brillouin zone.[6] The electronic convergence criterion was 1×10^{-7} Ha in energy difference. A Fermi-Dirac smearing of 0.2 eV width was used. The ionic convergence criteria were 1×10^{-6} Ha in energy difference or 2×10^{-4} Ha/Bohr in root-mean-squared force. The CANDLE solvation model was employed.[7] Unless stated otherwise, we used the solvent dielectric constant of 78 and a Debye length of 3 Å (i.e., a 1 M NaF concentration).

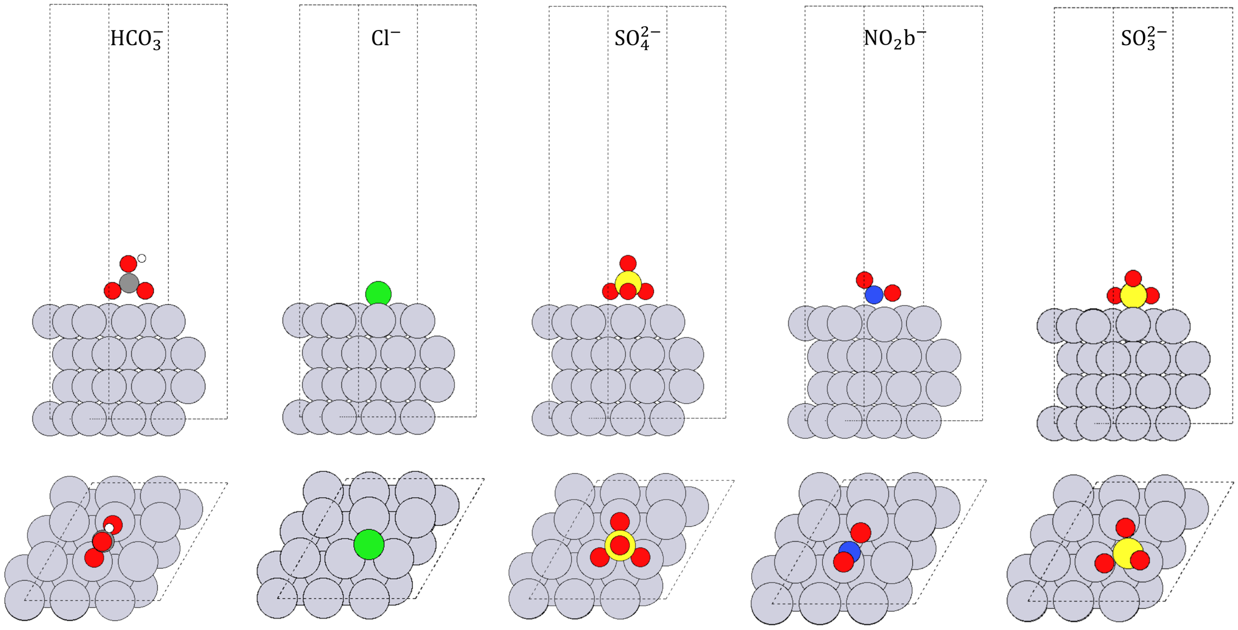

Figure S1: Side and top views of the slab model of a Pt(111) surface, adsorbed with HCO_3^-, Cl^-, SO_4^{2-}, NO_2b^-, and SO_3^{2-}.

The (111) surfaces of metals were constructed as a periodic 3×3×4 slab (Fig. S1 for Pt(111)). The bottom two layers are frozen during geometric optimization. The fcc lattice parameters were optimized in DFT for each considered exchange-correlation functional.

The anion adsorbates were placed on high symmetry sites on the (111) surfaces. Halides occupy the fcc hollow sites.[8] Oxo-anions with less than four oxygens (carboxylates, nitrate, sulfites, carbonates) adsorbs on two atop sites;[9] those with four oxygens (sulfates, phosphates, perchlorate) adsorb on three top sites.[10] Nitrite (NO_2^-) can either adsorb with two oxygens (denoted as NO_2a^-) or with oxygen and nitrogen (NO_2b^-, see Fig. S1).[11] Sulfite (SO_3^{2-}) adsorbs with one S atom.[12] These adsorption sites inform the parameter s_i in the Langmuir model (Section S6).

The grand free energies Ω for surface species are:

$$\Omega(U) = E_0 + E_{\text{ZPE}} - TS_{\text{vib}} - \varepsilon_F \times n_e \tag{S1}$$

The applied potential U on the SHE scale is converted as $U = (\varepsilon_F - \varepsilon_{\text{ref}})/e$ where ε_F is the Fermi energy and ε_{ref} is 4.66 eV as calibrated for the CANDLE solvation model.[7] The Helmholtz free energy F of surface species was approximated as the ground-state energy E_0 plus vibrational zero-point energy (E_{ZPE}) and entropic corrections (TS_{vib}). The vibrational frequencies are computed once without an applied potential (i.e., at the potential of zero charge) and assumed to be potential-independent. The frequencies of soft vibrational modes from frustrated translation/rotation are rounded to 50 cm^{-1}.

The Gibbs free energies of gas-phase species are:

$$G = E_0 + E_{\text{ZPE}} + k_BT + (H_{\text{trans}} + H_{\text{rot}} + H_{\text{vib}}) - T\left(S_{\text{trans}} + S_{\text{rot}} + S_{\text{vib}}\right) \tag{S2}$$

The k_BT term accounts for the pressure-volume contribution under the ideal gas assumption. Derivations for the enthalpic (H) and entropic (S) correction terms are outlined elsewhere.[13] We used the thermochemistry modules in Atomic Simulation Environment[14] for these corrections, which by default uses a standard ideal-gas-concentration at 298 K and 1 atm pressure (0.041 M). When necessary for thermodynamic cycles, corrections to different standard concentrations were added as $k_BT \ln(c/0.041)$ where c is the new standard concentration.

S1.2 Derivation of thermodynamic cycles

Here, we derive the thermodynamic cycles used for computing adsorption free energies of different anions. The anions are divided into groups depending on how their thermodynamic cycles are derived: Group I follows the protonation path, Group II follows the redox path, and Group III follows the path with both protonation and redox steps.

Group I: Protonation path

The simplest case is monoprotic anions: nitrate (NO_3^-), nitrite (NO_2^-), carboxylates ($RCOO^-$), and perchlorate (ClO_4^-), which follow the thermodynamic cycle shown in Fig. S2.

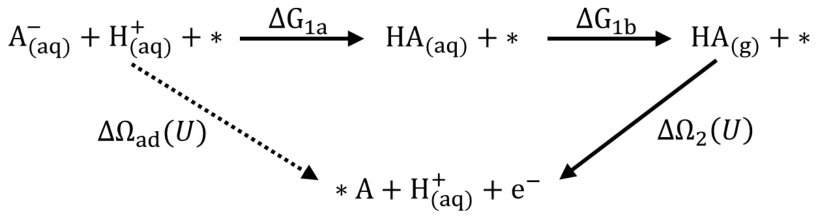

Figure S2: Thermodynamic cycle for monoprotic anions in group I. A are NO_3, NO_2, RCOO, or ClO_4.

$$\Delta G_{1a} = -k_B T \ln(10) \times \text{pKa} \tag{S3}$$

$$\Delta G_{1b} = k_B T \ln(k_B T \times H_{cp}) \tag{S4}$$

$$\Delta\Omega_2(U) = \Omega_{*A}(U) - \Omega_*(U) - G^{\ominus}_{HA(g)} + \frac{1}{2}G^o_{H_2(g)} - eU \tag{S5}$$

$$\Delta\Omega_{ad}(U) = \Delta G_{1a} + \Delta G_{1b} + \Delta\Omega_2(U) \tag{S6}$$

By definition, the Gibbs free energies G are not potential-dependent, while the electronic grand free energies $\Omega(U)$ are. The Boltzmann constant is k_B, and temperature T of 298 K is used throughout.

Step 1a represents aqueous-phase proton association and step 1b represents dehydration, which require the concentration-pressure Henry's constant (H_{cp}) and pKa value, respectively. Literature values of H_{cp} and pKa are shown in Table S1.[15–17] In step 2, the free energy of the aqueous proton plus electron (H^+(aq) + e^-) is equal to that of $\frac{1}{2}H_2(g) - eU$ in SHE scales.

Next, two diprotic anions follow the protonation path: (bi)carbonate (HCO_3^-/CO_3^{2-}), and (bi)sulfite (HSO_3^-/SO_3^{2-}), which has two acid dissociation steps (Fig. S3). The first dissociation step is equilibrated directly to aqueous CO_2(aq) or SO_2(aq), using the "apparent" pKa_1^{app}. Oxalic acid ($COOHCOO^-/COOCOO^{2-}$) follows a similar path but with "true" pKas.

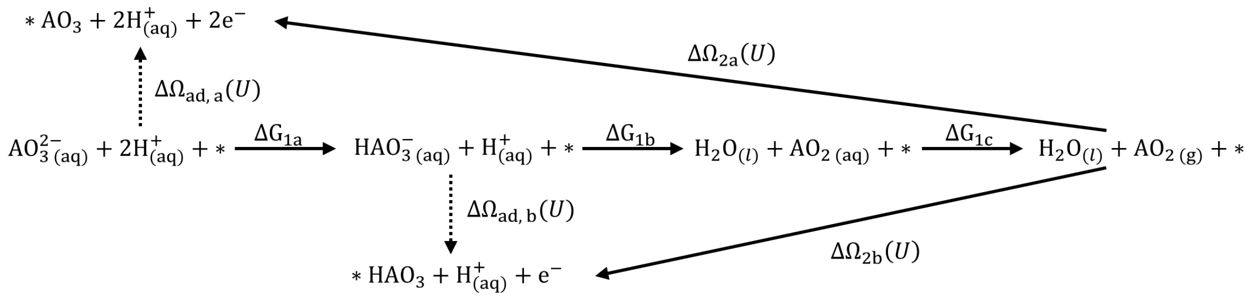

Figure S3: Thermodynamic cycles for diprotic anions in group I. A are C or S.

$$\Delta G_{1a} = -k_B T \ln(10) \times pKa_2 \tag{S7}$$

$$\Delta G_{1b} = -k_B T \ln(10) \times pKa_1^{app} \tag{S8}$$

$$\Delta G_{1c} = k_B T \ln(k_B T \times H_{cp}) \tag{S9}$$

$$\Delta\Omega_{2a}(U) = \Omega_{*AO_3}(U) - \Omega_*(U) - G^{\ominus}_{AO_2(g)} - G^{l}_{H_2O(l)} + G^{o}_{H_2(g)} - 2eU \tag{S10}$$

$$\Delta\Omega_{2b}(U) = \Omega_{*HAO_3}(U) - \Omega_*(U) - G^{\ominus}_{AO_2(g)} - G^{l}_{H_2O(l)} + \frac{1}{2}G^{o}_{H_2(g)} - eU \tag{S11}$$

$$\Delta\Omega_{ad,a}(U) = \Delta G_{1a} + \Delta G_{1b} + \Delta G_{1c} + \Delta\Omega_{2a}(U) \tag{S12}$$

$$\Delta\Omega_{ad,b}(U) = \Delta G_{1b} + \Delta G_{1c} + \Delta\Omega_{2b}(U) \tag{S13}$$

Consistent standard states are crucial. The final $\Delta\Omega_{ad}(U)$ are standardized to 1 M of anions and protons (i.e., pH = 0) in the aqueous-phase. This means the standard concentrations of gaseous species (i.e., HA(g) or AO_2(g)) must also be at 1 M, as denoted by $^{\ominus}$. On the SHE scales, the protons at standard concentration of 1 M are equilibrated to H_2(g) at 1 atm, as denoted by ^{o}. Finally, the standard state for H_2O(l) reflects pure water at 298 K, i.e., at a standard concentration of 55.34 M, as denoted by ^{l}. The Gibbs free energy of liquid water was computed from that of gaseous water (DFT) plus the experimental hydration free energy (-24.9 kJ/mol) and the standard state correction.

Group II: Redox path

This group consists of halides (F^-, Cl^-, and Br^-), which follow the cycle in Fig. S4.

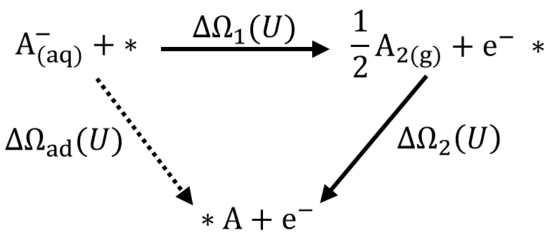

Figure S4: Thermodynamic cycle for halides. A are F, Cl, or Br.

$$\Delta\Omega_1(U) = e(U^{\text{red}} - U) \tag{S14}$$

$$\Delta\Omega_2(U) = \Omega_{*\text{A}}(U) - \Omega_*(U) - \frac{1}{2}G^o_{\text{A}_2(\text{g})} \tag{S15}$$

Step 1 represents the redox equilibrium between diatomic gaseous species and the aqueous anion. The experimental standard reduction potential U^{red} are publicly available and also given in Table S1.[18]

The standard states implicit to the standard redox potential are solution species at 1 M and gaseous species at 1 atm. Therefore, the standard Gibbs free energies of A_2(g) must be computed at 1 atm.

Group III: Protonation & redox combined path

Stepping up in complexity, (bi)sulfate (HSO_4^-/SO_4^{2-}) has both protonation and redox steps in the thermodynamic cycle.

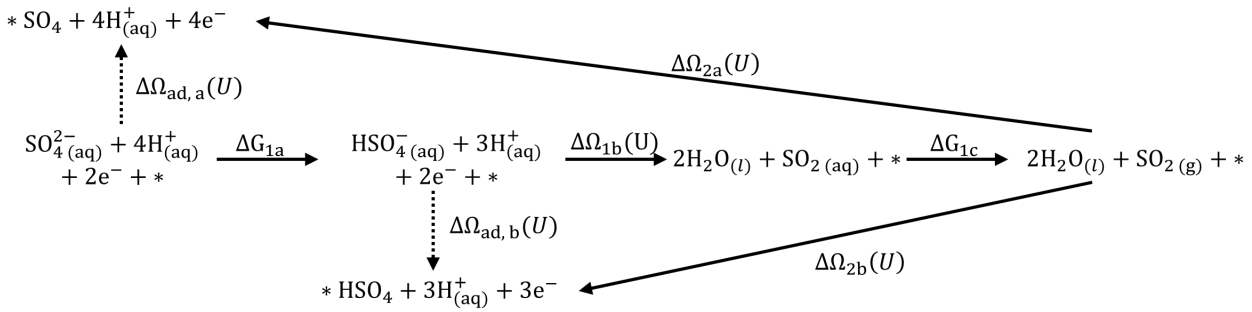

Figure S5: Thermodynamic cycle for (bi)sulfate.

$$\Delta G_{1a} = -k_B T \ln(10) \times \mathrm{pKa}_2 \tag{S16}$$

$$\Delta\Omega_{1b}(U) = 2e(U - U^{\mathrm{red}}) \tag{S17}$$

$$\Delta G_{1c} = k_B T \ln(k_B T \times H_{\mathrm{cp}}) \tag{S18}$$

$$\Delta\Omega_{2a}(U) = \Omega_{*\mathrm{SO_4}}(U) - \Omega_*(U) - G^{\ominus}_{\mathrm{SO_2(g)}} - 2G^{l}_{\mathrm{H_2O(l)}} + \frac{4}{2}G^{o}_{\mathrm{H_2(g)}} - 4eU \tag{S19}$$

$$\Delta\Omega_{2b}(U) = \Omega_{*\mathrm{HSO_4}}(U) - \Omega_*(U) - G^{\ominus}_{\mathrm{SO_2(g)}} - 2G^{l}_{\mathrm{H_2O(l)}} + \frac{3}{2}G^{o}_{\mathrm{H_2(g)}} - 3eU \tag{S20}$$

$$\Delta\Omega_{\mathrm{ad,a}}(U) = \Delta G_{1a} + \Delta\Omega_{1b}(U) + \Delta G_{1c} + \Delta\Omega_{2a}(U) \tag{S21}$$

$$\Delta\Omega_{\mathrm{ad,b}}(U) = \Delta\Omega_{1b}(U) + \Delta G_{1c} + \Delta\Omega_{2b}(U) \tag{S22}$$

Compared to (bi)sulfite (Fig. S3), (bi)sulfate has a redox equilibrium with SO_2(aq) (i.e., it involves a two-electrons transfer), making the reaction free energy potential-dependent (step 1b). The standard concentration for SO_2(g) must be 1 M, reflecting the redox equilibrium between HSO_4^-(aq) with aqueous SO_2(aq).

The thermodynamic cycle for ((di)hydrogen) phosphate ($H_2PO_4^-/HPO_4^{2-}/PO_4^{3-}$) mirrors that for sulfates with an additional proton dissociation step, and a redox equilibrium with PH_3(g) at 1 atm.

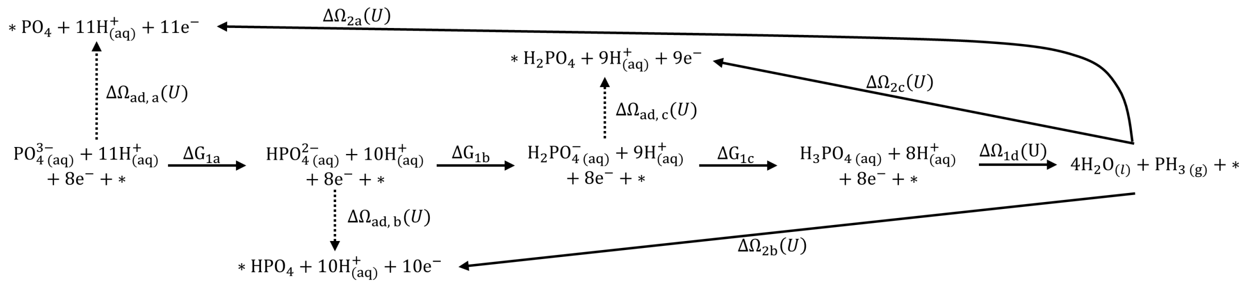

Figure S6: Thermodynamic cycle for ((di)hydrogen) phosphate.

$$\Delta G_{1a/b/c} = -k_B T \ln(10) \times pKa_{3/2/1} \tag{S23}$$

$$\Delta\Omega_{1d}(U) = 8e(U - U^{red}) \tag{S24}$$

$$\Delta\Omega_{2a}(U) = \Omega_{*PO_4}(U) - \Omega_*(U) - G^o_{PH_3(g)} - 4G^l_{H_2O(l)} + \frac{11}{2}G^o_{H_2(g)} - 11eU \tag{S25}$$

$$\Delta\Omega_{2b}(U) = \Omega_{*HPO_4}(U) - \Omega_*(U) - G^o_{PH_3(g)} - 4G^l_{H_2O(l)} + \frac{10}{2}G^o_{H_2(g)} - 10eU \tag{S26}$$

$$\Delta\Omega_{2c}(U) = \Omega_{*H_2PO_4}(U) - \Omega_*(U) - G^o_{PH_3(g)} - 4G^l_{H_2O(l)} + \frac{9}{2}G^o_{H_2(g)} - 9eU \tag{S27}$$

$$\Delta\Omega_{ad,a}(U) = \Delta G_{1a} + \Delta G_{1b} + \Delta G_{1c} + \Delta\Omega_{1d}(U) + \Delta\Omega_{2a}(U) \tag{S28}$$

$$\Delta\Omega_{ad,b}(U) = \Delta G_{1b} + \Delta G_{1c} + \Delta\Omega_{1d}(U) + \Delta\Omega_{2b}(U) \tag{S29}$$

$$\Delta\Omega_{ad,c}(U) = \Delta G_{1c} + \Delta\Omega_{1d}(U) + \Delta\Omega_{2c}(U) \tag{S30}$$

Proton electrosorption

We also computed proton electrosorption with GC-DFT and a redox-like thermodynamic cycle (i.e., the CHE model). The computation of $\Delta\Omega_{ad,H}(U)$ is as follows.

$$H^+ + e^- + * \xrightleftharpoons{\Delta\Omega_{ad,H}} *H$$

$$\Delta\Omega_{ad,H}(U) = \Omega_{*H}(U) - \Omega_*(U) - \frac{1}{2}G^o_{H_2(g)} + eU \tag{S31}$$

Table S1: Experimental values used for thermodynamic cycles. From left to right: pKa values for acid/conjugate base pairs; Henry's constants (H_{cp}) of the neutral acids; redox pairs and the standard reduction potential U^{red}.

Acid/Conjugate Base	pKa[a]	H_{cp} $\left(\frac{mol}{m^3 Pa}\right)$[b]	Redox pair	U^{red} (V vs. SHE)[c]
HNO_3/NO_3^-	-1.38	2.1×10^3	–	–
HNO_2/NO_2^-	3.35	4.8×10^{-1}	–	–
CH_3COOH/CH_3COO^-	4.76	4.0×10^1	–	–
$CH_2OHCOOH/CH_2OHCOO^-$	3.83	2.8×10^2	–	–
$CClH_2COOH/CClH_2COO^-$	2.87	1.1×10^3	–	–
CCl_2HCOOH/CCl_2HCOO^-	1.26	1.2×10^3	–	–
CCl_3COOH/CCl_3COO^-	0.51	7.3×10^2	–	–
CFH_2COOH/CFH_2COO^-	2.59	8.0×10^2	–	–
CF_2HCOOH/CF_2HCOO^-	1.34	3.0×10^2	–	–
CF_3COOH/CF_3COO^-	0.30	8.9×10^1	–	–
$HClO_4/ClO_4^-$	-10.00	9.9×10^3	–	–
$COOHCOOH/COOHCOO^-$	1.46	6.1×10^6	–	–
$COOHCOO^-/COOCOO^{2-}$	4.40	–	–	–
CO_2/HCO_3^- (apparent)	6.35[d]	3.4×10^{-4}	–	–
HCO_3^-/CO_3^{2-}	10.30	–	–	–
SO_2/HSO_3^- (apparent)	1.90	1.3×10^{-2}	–	–
HSO_3^-/SO_3^{2-}	7.21	–	–	–
HF/F^-	3.20	–	$F_2(g)/F^-$	2.890
HCl/Cl^-	-6.30	–	$Cl_2(g)/Cl^-$	1.360
HBr/Br^-	-9.00	–	$Br_2(g)/Br^-$	1.094
HSO_4^-/SO_4^{2-}	1.99	–	$HSO_4^-/SO_2(aq)$	0.099
$H_3PO_4/H_2PO_4^-$	2.15	–	$H_3PO_4/PH_3(g)$	-0.269
$H_2PO_4^-/HPO_4^{2-}$	7.09	–	–	–
HPO_4^{2-}/PO_4^{3-}	12.32	–	–	–

[a] Values are from PubChem[16] and Ripin and Evans[15] unless otherwise stated.

[b] All values are from Sander[17].

[c] All values are from Bratsch[18].

[d] Ripin and Evans[15] states the pKa of H_2CO_3 to be 3.60, but the apparent pKa of CO_2 is 6.35.[19]

S1.3 Sensitivity analysis of parameters in GC-DFT model

S1.3.1 Goodness of fit of $\Delta\Omega_{\mathbf{ad}}$ vs. U

The linear fitting $\Delta\Omega_{\text{ad}}$ vs. U yields the electrosorption γ and U^0 parameters for different anion-metal pairs. Table S2 shows the goodness of the fit (R^2) and the standard errors of γ and U^0 across our whole dataset. The standard errors of γ and U^0 are well below the experimental calibration RMSE (0.12 V for U^0 and 0.21 e for γ), and within the systematic DFT error range. This justifies our choice of using only three data points ($\Delta\Omega_{\text{ad}}$ vs. U), as well as not using higher order fitting.

Table S2: R^2 values and standard errors of γ and U^0 statistics for linear fitting $\Delta\Omega_{\text{ad}}$ vs. U across 107 anion-metal pairs.

Statistical parameters	Min	Max	Mean	Stdev
R^2	0.9971	0.9999	0.9996	0.0006
$\delta\gamma$ (e)	0.0003	0.0869	0.0200	0.0212
δU^0 (V)	0.0003	0.0978	0.0225	0.0239

S1.3.2 Slab construction

We tested the sensitivty of electrosorption U^0 on the slab construction of the metal surface. Fig. S7 shows that different constructions yield variation in U^0 of less than 0.05 V, well within the standard DFT systematic errors, as well as the experimental calibration RMSE (0.12 V). We therefore used the 4 total with 2 frozen layers construction since it is the least computationally demanding.

Potential (V vs. SHE)	$\Delta\Omega_{ad}$ (eV)		
	4 layers with 2 frozen 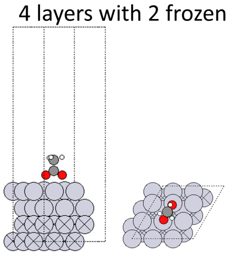	4 layers with 1 frozen 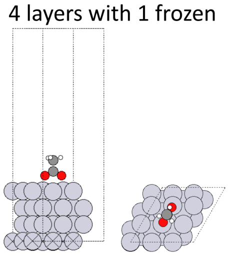	5 layers with 2 frozen 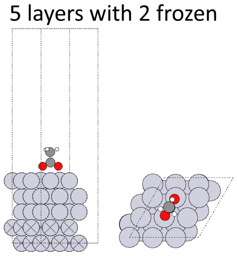
0.8	−0.913	−0.928	−0.940
0.4	−0.464	−0.476	−0.489
0	0.061	0.050	0.002
U^0 (V)	0.04	0.03	0.00

Figure S7: U^0 of CH_3COO^- on Pt(111) varying with surface slab construction.

S1.3.3 Coverage

Next, we examine the effects of varying coverages on U^0. At coverages $\leq$ 4/9 ML, U^0 varies insignificantly. We note that higher coverages (6/9 ML or 8/9 ML) could incur more significant changes, but were too computationally expensive to explore.

Potential (V vs. SHE)	$\Delta\Omega_{ad}$ (eV)		
	2/9 ML 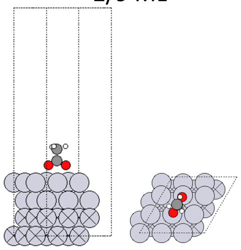	1/9 ML 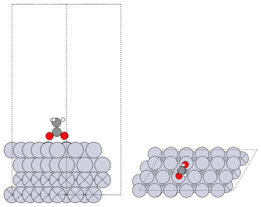	4/9 ML 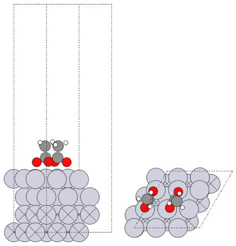
0.8	−0.913	−0.927	−0.920
0.4	−0.464	−0.478	−0.475
0	0.061	0.061	0.025
U^0 (V)	0.04	0.04	0.01

Figure S8: U^0 of CH_3COO^- on Pt(111) varying with surface coverages.

S1.3.4 Vibrational correction vs. potential

To save computational cost, we computed the vibrational frequencies of adsorbed anions at the potential of zero charge (PZC) and used those frequencies for thermal corrections (zero point energy and entropy) at all applied potentials. Table S9 show the vibrational zero-point energy varying little with the applied potential, justifying our model simplification.

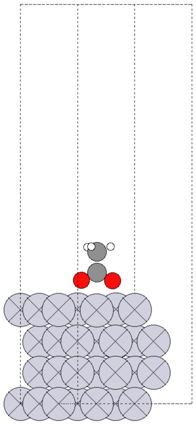

Potential (V vs. SHE)	ZPE (eV)	Δ (eV)
PZC	1.353	0.000
0.8	1.361	0.007
0.4	1.367	0.014
0	1.365	0.012

Figure S9: Vibrational zero-point energy (ZPE) of adsorbed CH_3COO on Pt(111) at various potentials.

S1.3.5 DFT functionals and implicit solvent dielectric constants

The computed U^0 and γ can be sensitive to the DFT functional and the electrolyte dielectric constant (ε_r) used in the CANDLE solvation model.

Here, we analyze the sensitivity of U^0 and γ for a subset of anions on Pt(111): Cl^-, OH^-, CH_3COO^-, CH_2OHCOO^-, CCl_2HCOO^-, HCO_3^-, and CO_3^{2-}. As seen in Fig. S10, the the RPBE functional consistently overpredicts U^0. That is, the binding of anions on Pt(111) is too weak compared to CV experimental results. The strengthened binding predicted by PBE-D3 over RPBE—particularly by Grimme's D3 Van der Waals correction—is well-documented in literature.[5,20–22]

In contrast, the effect of ε_r on U^0 is smaller. Suppressed dielectric constants (10 and 3) weaken the anion binding and thereby overpredict U^0 slightly. Finally, the sensitivity of γ is small to both functional and dielectric constant. In fact, using a suppressed dielectric constants slightly improve the γ RMSE, albeit for this very limited dataset.

We used the PBE-D3 functional and ϵ_r=78 throughout other parts of the work.

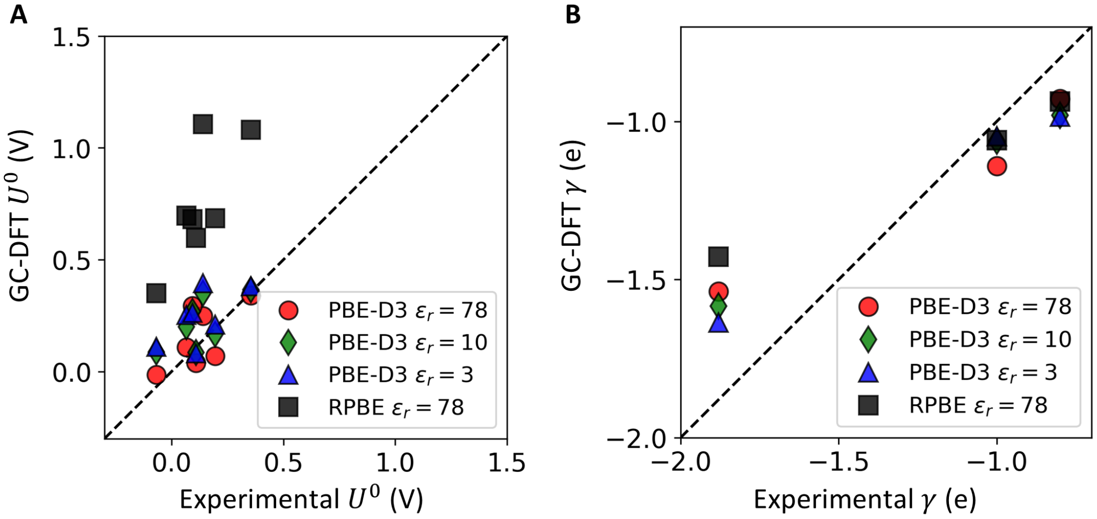

Figure S10: Sensitivity of (A) U^0 and (B) γ for a subset of anions on Pt(111). Comparing to experimental results, the U^0 RMSE are 0.177, 0.189, 0.207, and 0.665 V for PBE-D3 $\varepsilon_r = 78, 10, 3$ and RPBE $\varepsilon_r = 78$, respectively. γ RMSE in the same order are 0.226, 0204, 0.179, and 0.275 e.

S1.3.6 Micro-solvation with explicit water

The solvation of adsorbed anions may not be "sufficiently" described by an implicit solvation model. Here, we tested the effect of having an explicit water molecule solvating an adsorbed OH^- on Pt(111) (with implicit solvent still on).

We chose two intuitive configurations for the explicit water, either donating or accepting a hydrogen bond with the adsorbed OH^-, labeled as (A) and (B), respectively, in Fig. S11. These two final states shared one initial state, of which structure is a H_2O adsorbed without OH^- (not shown). For all states, we optimized the structures at their neutral charges, and then performed single-points at three applied potentials.

Fig. S11 indicates that the exact placement of explicit H_2O matters. Configuration A (H_2O accepting hydrogen bond from OH^-) yields very similar results (i.e., γ and U^0) with the no-water case, with U^0 in good agreement with experiment (-0.069 V) given the DFT functionals and implicit solvent used here. Configuration B (H_2O donating hydrogen bond to OH^-) yields much stronger binding for OH^-, shown by about -0.3 eV lower in U^0.

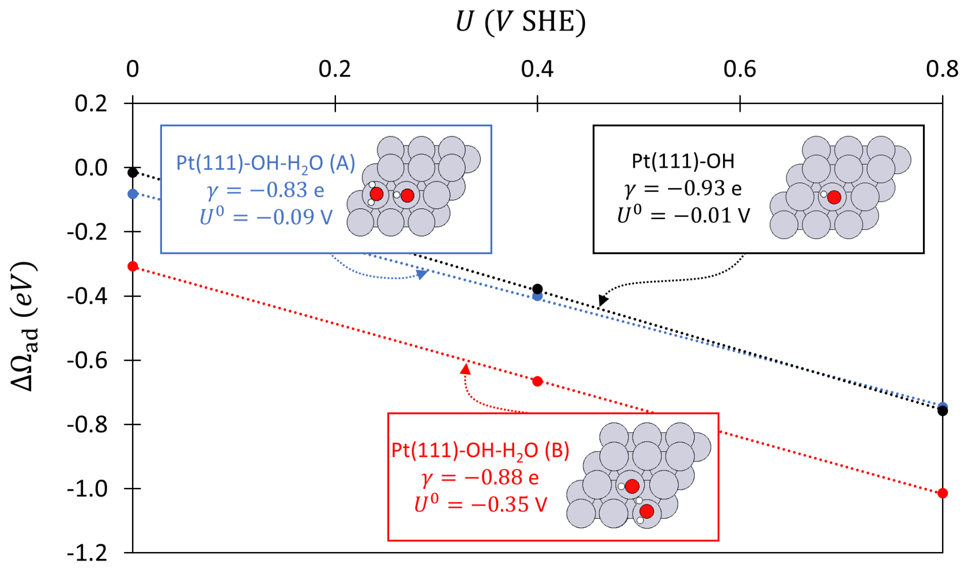

Figure S11: Adsorption free energy ($\Delta\Omega_{ad}$) of OH^- on Pt(111) with consideration of explicit micro-solvation. The black data points and fitted line are for without explicit water. The blue (A) and red (B) data points and fitted lines are for explicit water accepting and donating a hydrogen bond to adsorbed OH^-, respectively.

S2 Extracting results from experimental voltammograms

Here, we outline the procedure for extracting the standard equilibrium adsorption potential U^0 and the electrosorption valency γ of various anions on Pt(111) from experimental cyclic voltammograms (CVs). Table S3 shows the anions and corresponding references from which U^0 and γ were extracted.

Table S3: Experimental values of U^0 and γ for various anions and proton on Pt(111).

Ion	Reference	pH	Total conc. (M)	Ion conc. (M)	U^{peak} (V SHE)	U^0 (V SHE)	γ (e)
OH^-	Lipkowski[23]	1	55.3	1.00×10^{-13}	0.700	−0.069	−0.80
Cl^-	Lipkowski[24]	1	7.50×10^{-3}	7.50×10^{-3}	0.220	0.094	−1.00
Br^-	Lipkowski[25]	1	7.50×10^{-3}	7.50×10^{-3}	0.110	−0.016	−1.00
HSO_4^-	Feliu[26]	0.43	1.00×10^{-1}	9.69×10^{-2}	0.725	0.665	–
SO_4^{2-}	Feliu[26]	0.43	1.00×10^{-1}	3.13×10^{-3}	0.455	0.381	−1.80
HCO_3^-	Feliu[27]	1.56	3.40×10^{-2}	5.51×10^{-7}	0.510	0.140	−1.00
CO_3^-	Feliu[27]	1.56	3.40×10^{-2}	1.00×10^{-15}	0.510	0.067	−1.88
HPO_4^{2-}	Feliu[28]	1	1.00×10^{-4}	5.37×10^{-12}	0.559	0.226	−2.30
PO_4^{3-}	Feliu[28]	1	1.00×10^{-4}	2.57×10^{-23}	0.559	0.114	−2.30
CH_3COO^-	McCrum[9]	1	1.00×10^{-3}	1.74×10^{-7}	0.510	0.109	–
CH_2OHCOO^-	McCrum[9]	1	1.00×10^{-3}	1.48×10^{-6}	0.540	0.194	–
CCl_2HCOO^-	McCrum[9]	1	1.00×10^{-3}	3.55×10^{-4}	0.560	0.355	–
CF_3COO^-	McCrum[9]	1	1.00×10^{-3}	8.34×10^{-4}	0.570	0.387	–
ClO_4^-	Wright[29]	0	1.00	1.00	1.039	1.039	–
H^+	Lipkowski[23]	1	55.3	1.00×10^{-1}	0.200	0.141	+1.00

For each anion in the corresponding CV experiment, we first find the potential where current density peaks as the anion electrosorbs, i.e., U^{peak}. Assuming a coverage-independent electrosorption of anion (i.e., a Langmuir model), U^{peak} corresponds to the Langmuir equilibrium between anions in solution and electrosorbed anions.[30]

Next, we correct U^{peak} to the desired 1 M standard concentration of anion. This correction is essentially a Nernstian shift in the equilibrium potential from non-standard concentrations to the standard concentration:

$$U^0 = U^{\text{peak}} + \frac{RT}{nF} \ln \frac{c}{c^0} \tag{S32}$$

where R is the gas constant, F is the Faraday constant, n is the anion formal charge, c^0 is the anion standard concentration (i.e., 1 M), and c is the anion concentration at which U^{peak} is measured during a CV experiment. With known pH and total acid concentration used in CV experiments, acid-base equilibria are solved to obtain the ion concentration c, as shown in Table S3 (see section S6 for more details about solving acid-base equilibria).

In some references, additional analysis of the CV data yields the electrosorption valency γ from the slope of charge density versus Gibbs excess of adsorbed anions at a constant applied potential. These analyses sometimes yield γ varying with potential for some anions. In those cases (e.g., for SO_4^{2-}), we pick γ values at U^{peak} as the electrosorption valency of a given anion, and assume that γ is not potential-dependent. This assumption is sound, at the very least from our computational model, judging from Fig. 2 in the main text where the slope of adsorption free energy vs. potential (i.e., γ) is fairly constant across the potential range.

There are a few special considerations for HCO_3^-/CO_3^{2-}. We assigned a single CV's U^{peak} to both conjugated anions electrosorbing at roughly the same potential. This is supported by vibrational spectroscopic evidence showing both species present, reported by the same reference.[27] The total concentration of $CO_2/HCO_3^-/CO_3^{2-}$ is assumed to be the saturated concentration of CO_2 at 1 atm pressure, i.e., 0.034 M. Furthermore, there is a caveat in the electrosorption valency analysis, which only revealed that γ of CO_3^{2-} is $-0.88e$ lower than that of HCO_3^-. We then assumed γ of HCO_3^- to be $-1e$.

Similarly for $H_2PO_4^-/HPO_4^{2-}/PO_4^{3-}$, we initially assigned a single U^{peak} and a γ value to all three conjugated species. However, our theoretical values of U^0 and γ agree better with experimental values for HPO_4^{2-} and PO_4^{3-}, compared to $H_2PO_4^-$. This suggested that only HPO_4^{2-} and PO_4^{3-}, not $H_2PO_4^-$, may correspond to the electrosorption current observed in CV experiments (see Fig. 3 in the main text). Further discernment about which of HPO_4^{2-} or PO_4^{3-} are the more likely adsorbed species is outside the scope of our work. We, therefore, included both HPO_4^{2-} and PO_4^{3-} values in the experimental calibration for completeness.

S3 Anion-metal dataset

S3.1 Description and sources of features

Table S4: List of features with corresponding sources for anion and metal properties.

Symbols	Anion features	Sources
z	Anion formal charge	General chemistry
ΔG_{pKa}	Gibbs free energy of acid dissociation	Tabulated pKa[15,16]
E^{aH}_{HOMO}	HOMO energy when protonated	NWChem DFT
E^{a0}_{HOMO}	HOMO energy at neutral charge	NWChem DFT
E^{az}_{HOMO}	HOMO energy at $-z$ charge	NWChem DFT
E^{a1}_{HOMO}	HOMO energy at -1 charge	NWChem DFT
E^{a0}_{LUMO}	LUMO energy at neutral charge	NWChem DFT
E^{az}_{EA}	Electron affinity to create $-z$ charge	NWChem DFT
E^{a1}_{EA}	Electron affinity to create -1 charge	NWChem DFT
ΔE^{dis}_{H}	Energy of homolytic proton dissociation	NWChem DFT
μ_z	Surface-normal dipole moment in adsorbed geometry	VASP DFT
**Symbols**	**Metal features**	**Sources**
$\alpha_m^{1/3}$	Cubic root of metal atomic polarizability	Tabulated[31]
r_{vdw}	Metal atomic van der Waals radius	Tabulated[31]
E^{m}_{EA}	Metal atomic electron affinity	Tabulated[31]
V^2_{ad}	Square of coupling strength with O 2p relative to Cu	Tabulated[32]
a_{fcc}	Face-centered cubic lattice parameter	JDFTx DFT
ε^{v}_{F}	Fermi energy of (111) slab in vacuum	JDFTx DFT
ε_F	Fermi energy of (111) slab in implicit electrolyte	JDFTx DFT
W_d	Width of d-band of (111) slab	JDFTx DFT
ε_d	Center of d-band of (111) slab	JDFTx DFT
W_{sp}	Width of sp-band of (111) slab	JDFTx DFT
ε_{sp}	Center of sp-band of (111) slab	JDFTx DFT

To obtain anion features, we performed molecular DFT calculations in NWChem with the aug-cc-pVTZ basis set and the PBE functional. VASP calculations was performed to obtain μ_z, since VASP conveniently outputs the directional dipole moments while JDFTx does not. Optimized structures from JDFTx (in implicit solvent, no applied potential) was converted to VASP and ran as a single-point calculation with the same PBE-D3 functional.

S3.2 Feature correlation matrix

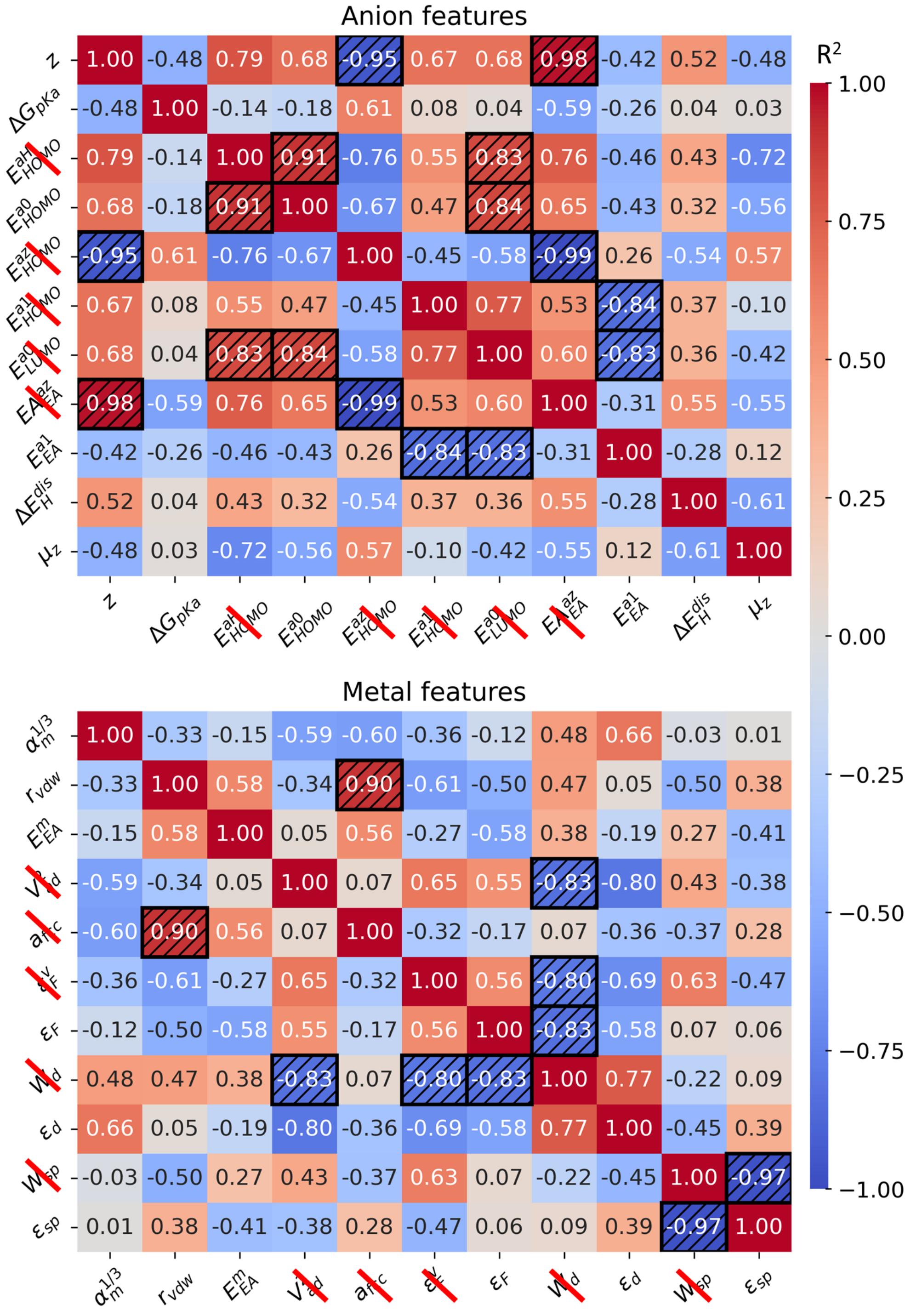

Figure S12: Pearson correlation R^2 matrix for anion features (top) and metal features (bottom). Pair of features with R^2 larger than 0.8 are boxed with diagonal hatches. One feature of each correlated pair is removed from subsequent model fitting, indicated by red slashes in axes labels.

S4 Symbolic regression model

To explore non-linear regression of features for U^0 and γ prediction, we systematically tested the SISSO model.[33] In brief, this symbolic regression model enumerate possible combinations of features and operators $\{+; -; \times; \div\}$, and iterate through the combinations to find an analytical form of the features that predict the labels (i.e., U^0 or γ) with the lowest test-set-RMSE.

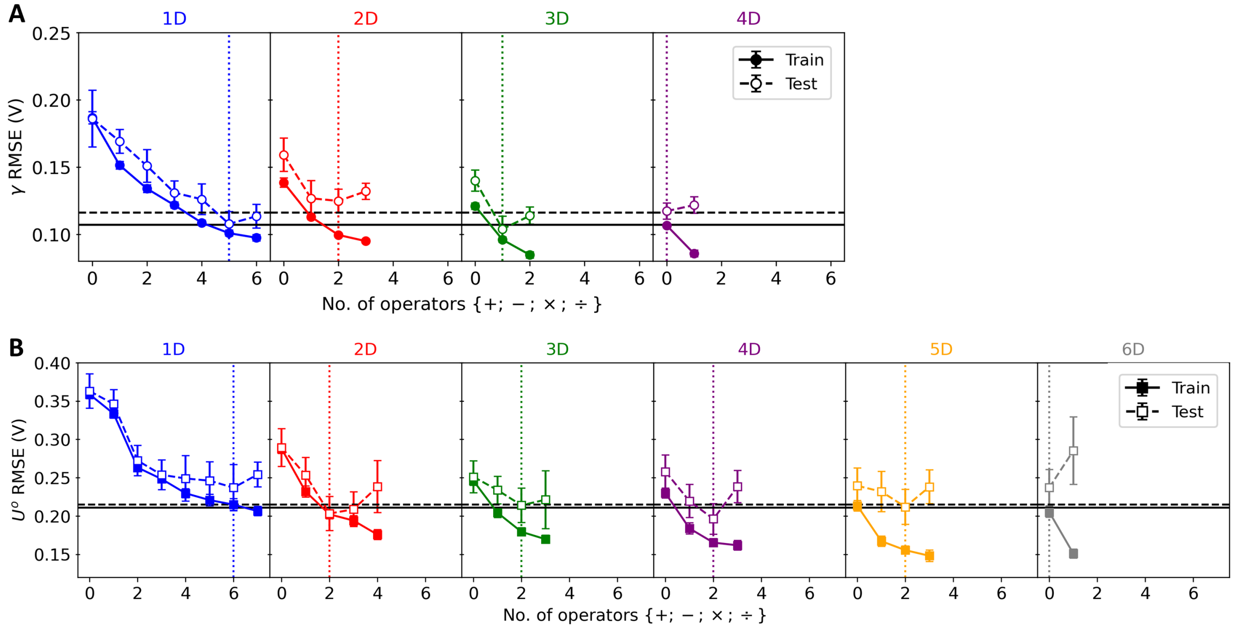

Figure S13: Hyperparameter tests of SISSO symbolic regression for predicting (A) γ and (B) U^0. Hyperparameters include the number of dimensions (nD) and the number of operators $\{+; -; \times; \div\}$ in each dimension (x-axis). RMSE averages and error bars are obtained from a five-fold cross validation. Training and testing RMSE are shown with filled and open symbols, respectively. Vertical dotted line indicate optimal model complexity before overfitting, i.e., when testing RMSE increases while training RMSE decreases. For comparison, horizontal dashed and solid black lines are training and testing RMSE, respectively, from MLR models using four features for γ and six features for U^0.

Hyperparameter tests (Fig. S13) revealed that adding nonlinear operators does not significantly improve model performance compared to the optimized MLR models. Note that the MLR model is simply the SISSO model with zero operators.

In addition, the SISSO model can be useful to detect "hidden" nonlinear relationship between features and labels. However, the optimal analytical equations (i.e., before overfitting) allows for little physical interpretation, at least to our knowledge (see Table S5).

Table S5: SISSO-determined equations for predicting γ and U^0. At each dimension, the number of operators $+$; $-$; $\times$; $\div$ is chosen to be right before overfitting (see Fig. S13). Some equations vary for different cross validation folds; only the equation giving the lowest testing RMSE is shown. Definitions of features are shown in Table S4. Fitted parameters include weights $\{a_i\}$ and intercept b.

nD	No. of operators	γ equations
1	5	$a_1(\varepsilon_d - \varepsilon_F - \varepsilon_{sp})\left(\frac{\mu_z}{\alpha_m^{1/3}} + z\right) + b$
2	2	$a_1(z\alpha_m^{1/3} + \mu_z) + a_2\frac{E^{a0}_{HOMO}}{\varepsilon_F\varepsilon_F} + b$
3	1	$a_1(\varepsilon_F z) + a_2(\varepsilon_d - \varepsilon_{sp}) + a_3(\varepsilon_F\mu_z) + b$
4	0	$a_1 z + a_2\mu_z + a_3\varepsilon_d + a_4\varepsilon_F + b$
nD	No. of operators	U^0 equations
1	6	$a_1\left(\frac{E^{a0}_{HOMO}+\Delta G_{pKa}}{\alpha_m^{1/3}} + \frac{\Delta E^{dis}_H E^m_{EA}}{\varepsilon_{sp}\alpha_m^{1/3}}\right) + b$
2	2	$a_1\frac{E^{a1}_{EA}E^m_{EA}}{z} + a_2\frac{E^{a0}_{HOMO}r_{vdw}}{\alpha_m^{1/3}} + b$
3	2	$a_1\frac{E^{a1}_{EA}E^m_{EA}}{z} + a_2\frac{E^{a0}_{HOMO}-\Delta G_{pKa}}{\alpha_m^{1/3}} + a_3\frac{E^{a1}_{EA}+\varepsilon_F}{\Delta G_{pKa}} + b$
4	2	$a_1\frac{E^{a1}_{EA}E^m_{EA}}{z} + a_2\frac{E^{a0}_{HOMO}r_{vdw}}{\alpha_m^{1/3}} + a_3\frac{E^{a1}_{EA}+\varepsilon_F}{\Delta G_{pKa}} + a_4\frac{\Delta G_{pKa}}{E^{a1}_{EA}\varepsilon_F} + b$
5	2	$a_1\frac{E^{a1}_{BA}E^m_{BA}}{z} + a_2\frac{E^{a0}_{HOMO}r_{vdw}}{\alpha_m^{1/3}} + a_3\frac{E^{a1}_{EA}+\varepsilon_F}{\Delta G_{pKa}} + a_4\frac{\Delta G_{pKa}}{E^{a1}_{EA}\varepsilon_F} + a_5\frac{\Delta G_{pKa}}{E^{a1}_{EA}-\Delta E^{dis}_H} + b$
6	0	$a_1 E^m_{EA} + a_2\alpha_m^{1/3} + a_3 E^{a0}_{HOMO} + a_4 E^{a1}_{EA} + a_5\Delta G_{pKa} + a_6\Delta E^{dis}_H + b$

In summary, while the SISSO models can allow for small improvements in prediction RMSE, their lack of physical interpretability prompts us to stick with the MLR models.

S5 Multiple linear regression (MLR) model

S5.1 MLR results for U^0

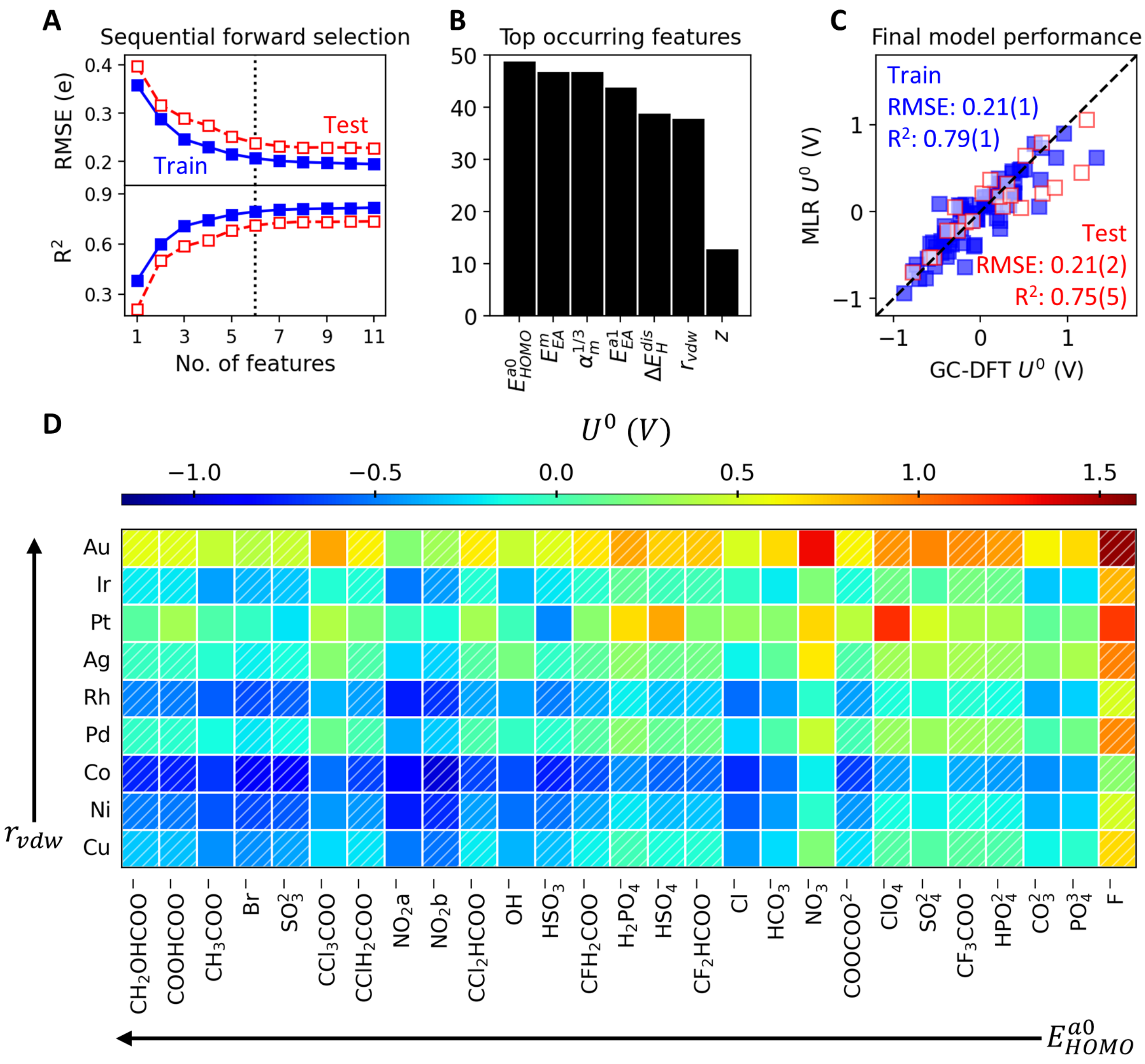

Figure S14: (A) Sequential forward selection applied to the U^0−MLR model, yielding six as the optimal number of features (dotted vertical line) where RMSE and R^2 no longer improves significantly with more features. (B) Histograms to count the most occurring features from 50 iterations of training the U^0−MLR model with six features on different 80:20 train-test splits. (C) Parity plot for the final U^0−MLR model trained on the six most occurring features. 5-fold cross validation metrics (RMSE and R^2) are shown as averages with standard errors in brackets. (D) Dataset matrix with U^0 values computed from GC-DFT (solid fill) or from the optimized U^0−MLR model (diagonal hatch). The matrix is ordered by increasing E^{a0}_{HOMO} for the anions and r_{vdw} for the metals, following the arrow directions.

S5.2 Retained charge analysis

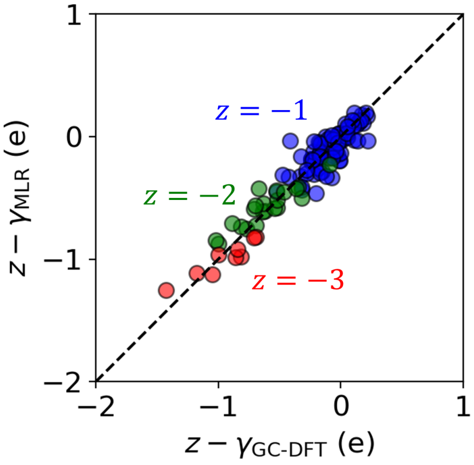

Figure S15: MLR-predicted versus GC-DFT-computed $z - \gamma$, resulting in the amount of charge retained on adsorbed anion. Different colors show different valency groups.

S5.3 Anion MLR equations

Final MLR equations for anion electrosorption trained all available data:

$$\gamma_{\mathrm{MLR}} = 0.608z + 0.394\mu_z + 0.221\varepsilon_F - 0.067\varepsilon_d \tag{S33}$$

$$U^0_{\mathrm{MLR}} = 0.349E^m_{EA} - 1.316\alpha_m^{1/3} + 1.057r_{vdw} + 0.185\Delta E^{dis}_H - 0.238E^{a0}_{HOMO} + 0.094E^{a1}_{EA} \tag{S34}$$

S6 Potential-dependent Langmuir model

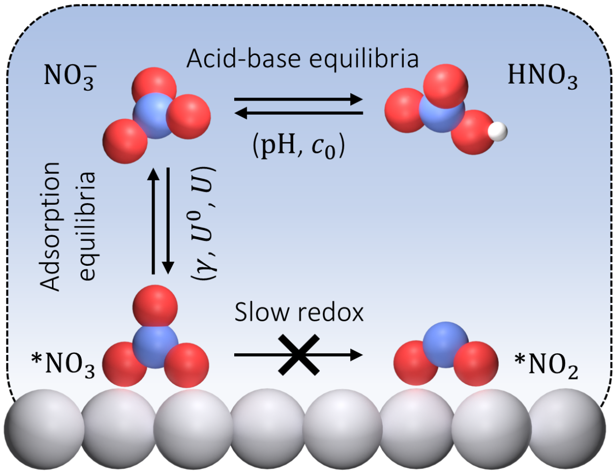

Figure S16: Schematic for the potential-dependent Langmuir model shown for NO_3^- as an example. The acid-base equilibria are solved by knowing the constant pH and total acid concentration c_0. The adsorption equilibria are solved by knowing the electrosorption valency γ, the adsorption equilibrium potential U^0 for any given anion-metal pairs, and the applied potential U. Any electron-transferred redox reactions are presumably slow and omitted.

The model construction starts with the acid-base equilibria, then the Langmuir adsorption equilibria (Fig. S16). We consider acid-base equilibria for any acid with n possible proton dissociation steps ($1 \leq n \leq 3$ for our dataset). The pH and total acid concentration c_0 are parameters, and the pKas of any given acids are known constants. Eqn. S35 depicts a system of linear equations to solve for the concentration of each conjugate species (denoted by the square brackets). $m \in \{0, 1, ..., n\}$ denotes the mth step of dissociation.

$$\mathrm{H_nA_{(aq)}} \xrightleftharpoons{\mathrm{Ka_1}} \mathrm{H^+_{(aq)}} + \mathrm{H_{n-1}A^-_{(aq)}} \cdots \xrightleftharpoons{\mathrm{Ka_m}} \mathrm{mH^+_{(aq)}} + \mathrm{H_{n-m}A^{m-}_{(aq)}}$$

$$\begin{cases} [\mathrm{H}_{n-m-1}\mathrm{A}^{-m-1}] = [\mathrm{H}_{n-m}\mathrm{A}^{-m}]10^{\mathrm{pH-pKa_m}} \\ \sum_{m=0}^{n} [\mathrm{H}_{n-m}\mathrm{A}^{-m}] = c_0 \end{cases} \qquad m \in \{0, 1, ..., n\} \tag{S35}$$

Next, the Langmuir adsorption equilibria consider anions electrosorbing on identical surface sites and having no lateral interactions (i.e., no coverage dependence). Eqn. S36 shows a system a non-linear equations to solve for the potential-dependent coverages $\theta_i(U)$ of each anion $i \in \{A, B, ...\}$.

$$\mathrm{A^{a-}} + * \xrightleftharpoons{\mathrm{K_A(U)}} *\mathrm{A} + \mathrm{a}e^- \;;\; \mathrm{B^{b-}} + * \xrightleftharpoons{\mathrm{K_B(U)}} *\mathrm{B} + \mathrm{b}e^- \;; ...$$

$$\begin{cases} \theta_i(U) = K_i(U)\theta_*^{s_i}(U)c_i \\ 1 = \theta_*(U) + \sum_i \theta_i(U) \end{cases} \quad i \in \{A, B, ...\} \tag{S36}$$

The free surface sites are denoted as $*$ and the number of sites that a given anion i occupies is s_i (which is informed from the DFT optimized structures of adsorbed anions). The solution-phase concentrations c_i are determined from acid-base equilibria (square brackets in Eqn. S35). The potential-dependent adsorption equilibrium constants come from the grand free energy of adsorption: $K_i(U) = \exp\left[-\beta\Delta\Omega_{\mathrm{ad},i}(U)\right] = \exp\left[-\beta\gamma_i(U - U_i^0)\right]$ where β is $1/k_BT$, and γ_i and U_i^0 are the electrosorption parameters for anion i.

The adsorption equilibria (Eqn. S36) also applies to proton electrosorption, of which γ and U^0 are also computed with GC-DFT. The bulk concentration of proton is trivially obtained from the pre-defined solution pH.

Practical steps (python code available at https://github.com/bolton2710/anion-electrosorption):

1. Defining the anion-metal system parameters: System parameters include the set of anions with their conjugates $\{i\}$ and the total acid concentration c_0, the solution pH, and the number of sites on which each anion adsorbs $\{s_i\}$.

2. Invoking predictions from the GC-DFT model: The GC-DFT and thermodynamic cycles method then predicts the electrosorption valency γ_i and standard adsorption equilibrium potential U_i^0 for each anion i (and proton) on the chosen metal surface.

3. Running two numerical solvers: A first numerical linear solver for the acid-base equilibria (Eqn. S35) yields the solution-phase concentrations of anions $\{c_i\}$. A second non-linear solver for the adsorption equilibria (Eqn. S36) yields the anion coverages at any applied potential $\{\theta_i(U)\}$.

References

(1) Sundararaman, R.; Letchworth-Weaver, K.; Schwarz, K. A.; Gunceler, D.; Ozhabes, Y.; Arias, T. A. JDFTx: Software for joint density-functional theory. *SoftwareX* **2017**, *6*, 278–284.

(2) Perdew, J. P.; Chevary, J. A.; Vosko, S. H.; Jackson, K. A.; Pederson, M. R.; Singh, D. J.; Fiolhais, C. Atoms, molecules, solids, and surfaces: Applications of the generalized gradient approximation for exchange and correlation. *Physical Review B* **1992**, *46*, 6671–6687.

(3) Hammer, B.; Hansen, L. B.; Nørskov, J. K. Improved adsorption energetics within density-functional theory using revised Perdew-Burke-Ernzerhof functionals. *Physical Review B - Condensed Matter and Materials Physics* **1999**, *59*, 7413–7421.

(4) Garrity, K. F.; Bennett, J. W.; Rabe, K. M.; Vanderbilt, D. Pseudopotentials for high-throughput DFT calculations. *Computational Materials Science* **2014**, *81*, 446–452.

(5) Grimme, S.; Antony, J.; Ehrlich, S.; Krieg, H. A consistent and accurate ab initio parametrization of density functional dispersion correction (DFT-D) for the 94 elements H-Pu. *Journal of Chemical Physics* **2010**, *132*.

(6) Monkhorst, H. J.; Pack, J. D. Special points for Brillouin-zone integrations. *Physical Review B* **1976**, *13*, 5188–5192.

(7) Sundararaman, R.; Goddard, W. A. The charge-asymmetric nonlocally determined local-electric (CANDLE) solvation model. *The Journal of Chemical Physics* **2015**, *142*.

(8) McCrum, I. T.; Akhade, S. A.; Janik, M. J. Electrochemical specific adsorption of halides on Cu 111,100, and 211: A density functional theory study. *Electrochimica Acta* **2015**, *173*, 302–309.

(9) Hasan, M. H.; McCrum, I. T. pKa as a Predictive Descriptor for Electrochemical Anion Adsorption. *Angewandte Chemie International Edition* **2024**, *63*.

(10) Yeh, K. Y.; Restaino, N. A.; Esopi, M. R.; Maranas, J. K.; Janik, M. J. The adsorption of bisulfate and sulfate anions over a Pt(1 1 1) electrode: A first principle study of adsorption

configurations, vibrational frequencies and linear sweep voltammogram simulations. *Catalysis Today* **2013**, *202*, 20–35.

(11) Getman, R. B.; Schneider, W. F. DFT-based characterization of the multiple adsorption modes of nitrogen oxides on Pt(111). *Journal of Physical Chemistry C* **2007**, *111*, 389–397.

(12) Ungerer, M. J.; Van Sittert, C. G.; De Leeuw, N. H. Behavior of S, SO, and SO3on Pt (001), (011), and (111) surfaces: A DFT study. *Journal of Chemical Physics* **2021**, *154*.

(13) McQuarrie, A. D.; Simon, D. J. *Molecular Thermodynamics*; University Science Books, 1999.

(14) Hjorth Larsen, A. et al. The atomic simulation environment—a Python library for working with atoms. *Journal of Physics: Condensed Matter* **2017**, *29*, 273002.

(15) Ripin, D. H.; Evans, D. A. pKa's of Inorganics and Oxo-acids. 2024; `https://organicchemistrydata.org/hansreich/resources/pka/pka_data/evans_pKa_table.pdf`.

(16) Kim, S.; Chen, J.; Cheng, T.; Gindulyte, A.; He, J.; He, S.; Li, Q.; Shoemaker, B. A.; Thiessen, P. A.; Yu, B.; Zaslavsky, L.; Zhang, J.; Bolton, E. E. PubChem 2025 update. *Nucleic Acids Research* **2025**, *53*, D1516–D1525.

(17) Sander, R. Compilation of Henry's law constants (version 4.0) for water as solvent. *Atmospheric Chemistry and Physics* **2015**, *15*, 4399–4981.

(18) Bratsch, S. G. Standard Electrode Potentials and Temperature Coefficients in Water at 298.15 K. *Journal of Physical and Chemical Reference Data* **1989**, *18*, 1–21.

(19) Adamczyk, K.; Prémont-Schwarz, M.; Pines, D.; Pines, E.; Nibbering, E. T. Real-time observation of carbonic acid formation in aqueous solution. *Science* **2009**, *326*, 1690–1694.

(20) Göltl, F.; Murray, E. A.; Tacey, S. A.; Rangarajan, S.; Mavrikakis, M. Comparing the performance of density functionals in describing the adsorption of atoms and small molecules on Ni(111). *Surface Science* **2020**, *700*, 121675.

(21) Bartaquim, E. O.; Bezerra, R. C.; Bittencourt, A. F. B.; Da Silva, J. L. F. Computational investigation of van der Waals corrections in the adsorption properties of molecules on the Cu(111) surface. *Physical Chemistry Chemical Physics* **2022**, *24*, 20294–20302.

(22) Araujo, R. B.; Rodrigues, G. L.; dos Santos, E. C.; Pettersson, L. G. Adsorption energies on transition metal surfaces: towards an accurate and balanced description. *Nature Communications* **2022**, *13*, 1–14.

(23) Mostany, J.; Herrero, E.; Feliu, J. M.; Lipkowski, J. Determination of the Gibbs excess of H and OH adsorbed at a Pt(1 1 1) electrode surface using a thermodynamic method. *Journal of Electroanalytical Chemistry* **2003**, *558*, 19–24.

(24) Garcia-Araez, N.; Climent, V.; Herrero, E.; Feliu, J.; Lipkowski, J. Thermodynamic studies of chloride adsorption at the Pt(1 1 1) electrode surface from 0.1 M HClO4 solution. *Journal of Electroanalytical Chemistry* **2005**, *576*, 33–41.

(25) Garcia-Araez, N.; Climent, V.; Herrero, E.; Feliu, J.; Lipkowski, J. Thermodynamic studies of bromide adsorption at the Pt(1 1 1) electrode surface perchloric acid solutions: Comparison with other anions. *Journal of Electroanalytical Chemistry* **2006**, *591*, 149–158.

(26) Garcia-Araez, N.; Climent, V.; Rodriguez, P.; Feliu, J. M. Thermodynamic analysis of (bi)sulphate adsorption on a Pt(1 1 1) electrode as a function of pH. *Electrochimica Acta* **2008**, *53*, 6793–6806.

(27) Martínez-Hincapié, R.; Berná, A.; Rodes, A.; Climent, V.; Feliu, J. M. Surface Acid-Base Properties of Anion-Adsorbed Species at Pt(111) Electrode Surfaces in Contact with CO2-Containing Perchloric Acid Solutions. *Journal of Physical Chemistry C* **2016**, *120*, 16191–16199.

(28) Mostany, J.; Martínez, P.; Climent, V.; Herrero, E.; Feliu, J. M. Thermodynamic studies of phosphate adsorption on Pt(1 1 1) electrode surfaces in perchloric acid solutions. *Electrochimica Acta* **2009**, *54*, 5836–5843.

(29) Attard, G. A.; Brew, A.; Hunter, K.; Sharman, J.; Wright, E. Specific adsorption of perchlorate anions on Pt{hkl} single crystal electrodes. *Physical Chemistry Chemical Physics* **2014**, *16*, 13689–13698.

(30) Li, Y.; Janik, M. J. Recent progress on first-principles simulations of voltammograms. *Current Opinion in Electrochemistry* **2019**, *14*, 124–132.

(31) Rumble, J. R. *CRC Handbook of Chemistry and Physics 105th Edition*, 105th ed.; CRC Press/Taylor and Francis: Boca Raton, FL, 2023.

(32) Ruban, A.; Hammer, B.; Stoltze, P.; Skriver, H.; Nørskov, J. Surface electronic structure and reactivity of transition and noble metals. *Journal of Molecular Catalysis A: Chemical* **1997**, *115*, 421–429.

(33) Ouyang, R.; Curtarolo, S.; Ahmetcik, E.; Scheffler, M.; Ghiringhelli, L. M. SISSO: A compressed-sensing method for identifying the best low-dimensional descriptor in an immensity of offered candidates. *Physical Review Materials* **2018**, *2*, 1–11.
